# Distinct cellular and junctional dynamics independently regulate the rotation and elongation of the embryonic gut in *Drosophila*

**DOI:** 10.1371/journal.pgen.1011422

**Published:** 2024-10-07

**Authors:** Mikiko Inaki, Takamasa Higashi, Satoru Okuda, Kenji Matsuno

**Affiliations:** 1 Department of Life Sciences, Graduate School of Science, University of Hyogo, Hyogo, Japan; 2 Department of Biological Sciences, Graduate School of Science, The University of Osaka, Toyonaka, Osaka, Japan; 3 Nano Life Science Institute, Kanazawa University, Kanazawa, Japan; University of Michigan, UNITED STATES OF AMERICA

## Abstract

Complex organ structures are formed with high reproducibility. To achieve such intricate morphologies, the responsible epithelium undergoes multiple simultaneous shape changes, such as elongation and folding. However, these changes have typically been assessed separately. In this study, we revealed how distinct shape changes are controlled during internal organ morphogenesis. The *Drosophila* embryonic hindgut undergoes left-right asymmetric rotation and anteroposterior elongation in a tissue-autonomous manner driven by cell sliding and convergent extension, respectively, in the hindgut epithelia. However, the regulation of these processes remains unclear. Through genetic analysis and live imaging, we demonstrated that cell sliding and convergent extension are independently regulated by Myosin1D and E-cadherin, and Par-3, respectively, whereas both require MyosinII activity. Using a mathematical model, we demonstrated that independently regulated cellular dynamics can simultaneously cause shape changes in a single mechanical system using anisotropic edge contraction. Our findings indicate that distinct cellular dynamics sharing a common apparatus can be independently and simultaneously controlled to form complex organ shapes. This suggests that such a mechanism may be a general strategy during complex tissue morphogenesis.

## Author summary

Complex organ structures are formed with high reproducibility. To achieve such intricate morphologies, the tissue undergoes multiple shape changes, such as elongation and folding, which are driven by distinct cellular behaviors. However, these changes have been assessed separately and the regulatory mechanisms are not fully understood. In this study, we explored the regulation of multiple tissue deformations using the *Drosophila* embryonic hindgut as a model system. The hindgut simultaneously undergoes left-right asymmetric rotation and anterior-posterior elongation, driven by cell sliding and intercalation, respectively. Through genetic analysis, live imaging, and mathematical modeling, we reveal that distinct genetic pathways regulate these processes, despite both utilizing common cell boundary contraction. Our findings indicate that distinct cellular dynamics sharing a common apparatus can be independently and simultaneously controlled to form complex organ shapes. These mechanisms might be widely applicable in the morphogenesis of complex organs.

## Introduction

Organ shapes are optimized to execute their functions [1]. The epithelium frequently plays a significant role in determining organ shape [2]. Therefore, the mechanisms underlying epithelial morphogenesis have been extensively studied and have been found to involve oriented cell division, directional cell migration, polarized cell deformation, and anisotropic cell rearrangement, among others [3–6]. Over the past decade, the cellular and molecular bases underlying each mechanism in epithelial morphogenesis have become well understood, although they have been studied separately in simplified systems. However, it is hypothesized that complex epithelial morphology results from the simultaneous and coordinated functioning of these morphogenetic mechanisms. This implies that the mechanical processes governing tissue morphogenesis can be delineated into distinct setups that collaboratively control the dynamic behaviors of a single tissue. Yet, the potential interactions or independence among these mechanisms remain unclear.

The *Drosophila* embryonic hindgut is an organ in which complex morphogenetic events occur simultaneously. The hindgut is composed of a monolayer epithelial tube and overlaying visceral muscles [7]. The embryonic hindgut is initially formed as a bilaterally symmetric structure, in which the anterior end curves toward the ventral side of the embryos at stage 12 [7–9]. Subsequently, at least two morphogenetic events, namely the elongation and left-handed rotation of the hindgut epithelial tube, concurrently occur at stage 13. Consequently, the hindgut has a left–right (LR) asymmetric hook-like structure [8–10].

The elongation of the hindgut epithelial tube is a typical example of convergent extension^10^. Similarly, convergent extension is involved in the elongation of the kidney and cochlea in vertebrates and the trachea in *Drosophila* [11–14]. It is also involved in various types of epithelial morphogenesis during embryogenesis, such as germ band extension in *Drosophila* and dorsal mesoderm extension in *Xenopus* and zebrafish [15,16]. During convergent extension, epithelial cells intercalate with each other toward the medial direction; this is referred to as cell intercalation and is driven by anisotropic cell junction remodeling [4]. Consequently, these tissues become narrower and longer, as observed in the elongation of the *Drosophila* embryonic hindgut [10]. Genes required for convergent extension have been identified by extensive genetic analyses in the *Drosophila* embryonic hindgut; however, their functions may not be specific to this process [17,18].

Another event in the morphogenesis of the *Drosophila* embryonic hindgut is its 90-degree counterclockwise rotation, as viewed from the posterior end [9]. Because of this rotation, the direction of bending at the anterior tip of the hindgut changes from ventral to right [9]. Before the rotation begins, the hindgut epithelial cells show chirality, manifesting as LR asymmetric tilt of their apical surfaces; this is known as cell chirality [9,19,20]. Dissolution of cell chirality consequently provides a driving force for hindgut rotation [9,21,22]. This idea was supported by the analyses of various mutations affecting cell chirality and LR asymmetry of the hindgut [9,21–24]. For example, in null mutants of *Drosophila Myosin 1D* (*Myo1D*), the LR asymmetry of various organs, including the rotation direction of the embryonic hindgut, is reversed [25,26]. *Myo1D*, also referred to as *Myosin 31DF*, encodes a class I myosin [25,26]. In *Myo1D* mutants, cell chirality is the mirror image of that noted in the wild type before hindgut rotation [9,25]. Furthermore, in mutants of *Drosophila E-cadherin* (*E-cad*), encoding an evolutionarily conserved epithelial cell adhesion protein, the randomization of hindgut LR asymmetry coincides with the depletion of cell chirality [9]. As an intermediate step between cell chirality and hindgut rotation, cell chirality is converted into cell sliding, a behavior of epithelial cells, which enables cells to change their relative position to sliding in one direction [22]. Chiral cell sliding is the ultimate step to drive the left-handed rotation of the hindgut [22].

Convergent extension and chiral cell sliding contribute simultaneously to the morphogenesis of the hindgut epithelium, making the hindgut an excellent system for studying the potential independence and relevance of these two morphogenetic mechanisms. This study employed live imaging analyses to assess the effects of genetic modifications on convergent extension and chiral cell sliding *in vivo*. Additionally, an organ culture system of the hindgut was developed, incorporating chemical inhibitors to quantitatively analyze both convergent extension and chiral cell sliding *ex vivo* at the whole-organ and single-cell levels. Furthermore, mathematical models of hindgut morphogenesis were constructed to evaluate the individual and integrated contributions of these mechanisms. Our analyses revealed that while convergent extension and chiral cell sliding are separable events, they share common machinery that is utilized differently in the polarized contraction of cell boundaries: anterior-posterior/distal–proximal anisotropy and chirality. We found that these mechanisms can independently and concurrently induce convergent extension and chiral cell sliding, respectively. Therefore, our results demonstrate that the concurrent actions of these distinct morphogenetic mechanisms are crucial for the complex transformation of organ shapes.

## Results

### LR defects associated with *E-cad* and *Myo1D* modifications coincided with cell sliding in the hindgut

During the LR asymmetric development of the embryonic hindgut, its epithelial tube concurrently rotates and elongates through cell sliding and convergent extension, respectively [22]. However, whether these two epithelial dynamics operate independently of each other or have mutual interdependence remains unclear. To distinguish between these two possibilities, we genetically altered the activities of *E-cad* and *Myo1D* that have been shown to play essential roles in the LR asymmetric development of the hindgut and then examined whether such genetic modifications affect either cell sliding or convergent extension or both.

**Fig 1:**
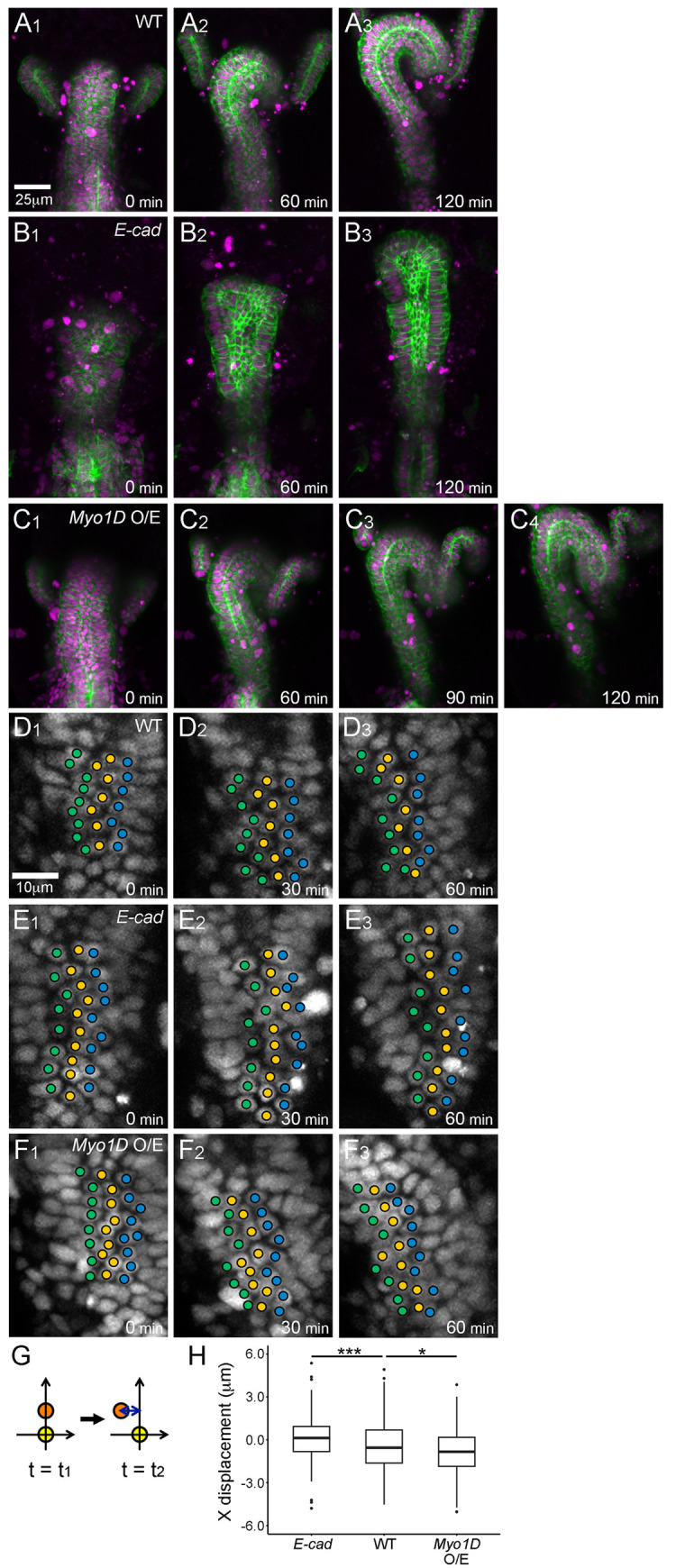
*E-cad* and *Myo1D* perturbation affected hindgut rotation and cell sliding. (A1–C4) Still shots from time-lapse Videos of the hindgut epithelium visualized using myr-GFP (membrane in green) and RedStinger (nuclei in magenta) from 0 to 120 min in the wild type (WT) (A1–A3), *E-cad* homozygote (B1–B3), and WT overexpressing *Myo1D* (*Myo1D* O/E) (C1–C4). (D1–F3) Displacement of nuclei (visualized by RedStinger) from 0 to 60 min in the hindgut epithelium of the WT (D1–D3), *E-cad* homozygote (E1–E3), and *Myo1D* O/E (F1–F3). Three central columns of nuclei are marked by green, yellow, and blue circles. (G) Schematic to quantify x displacement of a nucleus. The coordinates of two nuclei located along the anterior–posterior axis at two time points (t = t1 and t = t2) were observed. The subjacent nucleus was set at (0, 0), and the relative displacement of the upper nucleus in the x direction was measured every 30 min until 60 min. (H) Boxplots of x displacement in the *E-cad* mutant (n = 175, N = 7), WT (n = 164, N = 7), and *Myo1D* O/E (n = 180, N = 5). n and N in parentheses represent cell and embryo numbers, respectively. Black dots are outliers. ***p < 0.001. *p < 0.05. For all images, the anterior is on top. From A1 to F3, the time elapsed from the start of the Video is shown on the lower right. Scale bars in A1 and D1 are 25 and 10 μm, respectively.

We previously found that the LR asymmetry of the hindgut is mostly randomized in *E-cad*-mutant embryos at stage 14 [9]. Consistent with the previous idea that cell chirality of the hindgut epithelial cells drives the LR asymmetric rotation of the hindgut, cell chirality was found to be abolished in these *E-cad*-mutant cells [9]. In this study, to determine the roles of *E-cad* in cell sliding and convergent extension, we performed live imaging of the hindgut in an *E-cad*-null mutant, *shotgun*^*R69*^ (*shg*^*R69*^) [27]. UAS*-RedStinge*r (encoding a nuclear marker) and UAS*-myr-GFP* (encoding a membrane marker) were specifically expressed in the hindgut epithelium by the Gal4–UAS system [28–30]. From the dorsal side of the embryo, three-dimensional time-lapse images of the hindgut were taken for 120 min from late stage 12, which is sufficient time for the wild-type hindgut to complete its 90-degree rotation (Figs 1A1–3, S1 Video) [22]. However, the *E-cad*-mutant hindgut of the hardly rotated under this condition (Figs 1B1–3, S2 Video). To visually detect cell sliding, each column of nuclei that lined up along the anterior–posterior axis at 0 min was indicated by different colored circles (Fig 1D1). After 30 and 60 min, the relative positions of the nuclei did not change markedly in *E-cad*-mutant embryos (Fig 1E1–1E3 and S3 Video); however, the columns of nuclei slanted to the left in the wild type, as reported previously (Fig 1D1–1D3 and S4 Video) [22]. Thus, cell sliding was severely diminished in *E-cad*-mutant embryos.

To confirm this observation quantitatively, we measured relative cell displacement in the x direction, as described previously [22]. In brief, we set a subjacent (posterior in the hindgut) nuclear position at (0, 0) coordinates and measured the relative x displacement of the above nucleus against the subjacent nucleus every 30 min (Fig 1G). The wild-type hindgut epithelium had negative x displacement, showing leftward relative movement, as reported previously. In contrast, the *E-cad* epithelium hardly showed x displacement (Fig 1H) [22]. These results demonstrated that cell sliding requires *E-cad*.

*Myo1D* exhibits dextral activity to define the enantiomorphic states of organ and cell chirality [9,25,26]. Thus, organ and cell chirality become the mirror images of those noted in the wild-type counterparts in the absence of *Myo1D* [9,25,26]. Consistent with these findings, compared with the wild type, the direction of cell sliding is reversed in *Myo1D* mutants [22]. In contrast, the overexpression of wild-type *Myo1D* induces *de novo* chirality in organs and cells [25,31]. Therefore, in this study, we overexpressed wild-type *Myo1D* in the hindgut epithelium driven by *byn*-Gal4 and analyzed the potential reinforcement of cell sliding. As shown in Fig 1A, the wild-type hindgut took approximately 120 min to complete 90-degree rotation (Fig 1A1–1A3). However, upon the overexpression of *Myo1D*, the hindgut occasionally showed overrotation (six out of 13 embryos). Under this condition, the hindgut completed its 90-degree rotation in 90 min and continued to rotate further at least until 120 min (Fig 1C1–1C4, S5 Video).

We found that such acceleration of hindgut rotation coincided with an increase in cell sliding (Fig 1F and 1H). In the *Myo1D* overexpression condition, the columns of nuclei at 0 min gradually slanted to the left after 30 and 60 min; the degree of sliding was greater than that noted in the wild type (Fig 1F1–1F3 and S6 Video). This result was quantitatively supported by the result of relative cell displacement analysis, which revealed significantly more negative x displacement than that in the wild type (Fig 1H). These results suggest a positive correlation between the degree of cell sliding and the expression level of *Myo1D*. Based on the results of *E-cad* and *Myo1D* analyses, we established conditions to reduce and facilitate cell sliding, which could be used to assess whether cell sliding is coupled with convergent extension.

### *E-cad* and *Myo1D* did not affect convergent extension

**Fig 2:**
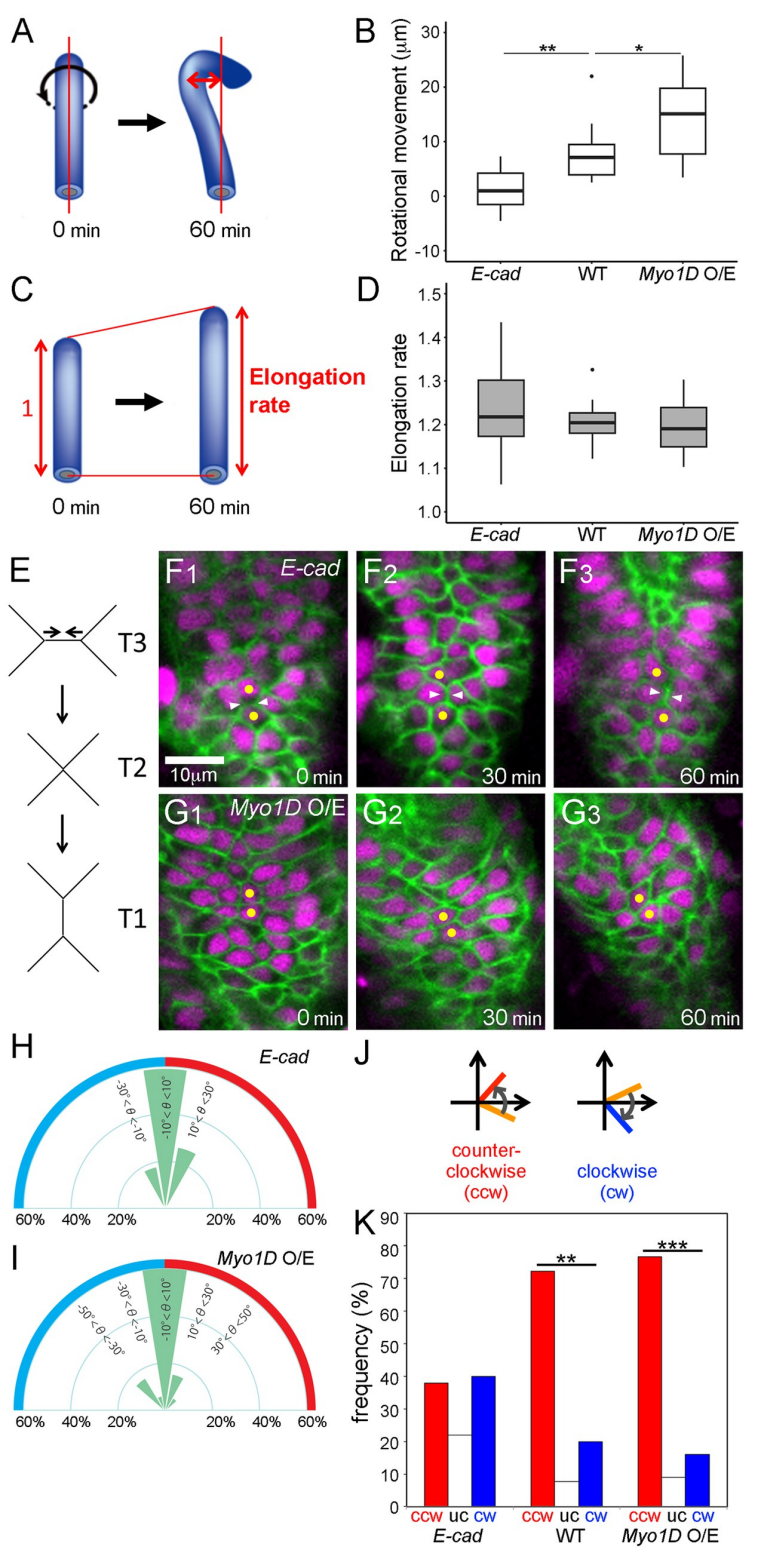
*E-cad* and *Myo1D* perturbation did not affect hindgut elongation and cell intercalation. (A–D) Quantification of hindgut deformation. (A) Schematic diagram of rotational movement index (indicated by a two-way arrow). (B) Boxplots of rotational movement index (μm) in the *E-cad* mutant (N = 10), wild type (N = 9), and wild type overexpressing *Myo1D* (N = 15). (C) Schematic diagram of elongation rate. The relative length of the hindgut at 60 min (indicated by a right two-way arrow) to its initial length (indicated by a left two-way arrow with “1”) was defined as the elongation rate. (D) Boxplots of elongation rate in the *E-cad* mutant (N = 10), wild type (N = 9), and wild type overexpressing *Myo1D* (N = 14). In b and d, black dots are outliers. **p < 0.01. *p < 0.05. (E) Schema of T3 to T1 transition. (F1–G3) Still shots from time-lapse Videos of cell nuclei (magenta) and boundaries (green) in the hindgut epithelium of the *E-cad* mutant (F1–F3) and wild type overexpressing *Myo1D* (G1–G3), visualized as described in Fig 1B and 1C, respectively. The time elapsed from the start of the Video is shown on the lower right. Yellow circles indicate cells next to each other in the first frame of the Video (F1–G3). A cell boundary showing T3 to T1 transition indicated by white arrowheads (F1–F3). The anterior is on top. (H, I) Frequencies of cell boundaries diminished during junction remodeling in the hindgut of the *E-cad* mutant (H) (n = 20, N = 4) and wild type overexpressing *Myo1D* (I) (n = 12, N = 5) with every 20-degree angle bin. (J, K) Angle change in the cell boundaries from 0 to 60 min. (J) Schemes for the analysis of cell boundary rotation. Initial cell boundaries (0 min) are shown in orange. Boundaries rotating counterclockwise (ccw) and clockwise (cw) are shown in red and blue, respectively. (K) Frequency of boundaries rotating ccw (red) and cw (blue) and remaining unchanged (uc, white) in the *E-cad* mutant (n = 50, N = 7), wild type (n = 55, N = 5), and wild type overexpressing *Myo1D* (n = 57, N = 5). n and N in parentheses represent cell and embryo numbers, respectively. ***p < 0.001. **p < 0.01.

Under the aforementioned conditions, we next analyzed whether cell sliding is concomitant with convergent extension in the hindgut epithelium. To quantitatively analyze the degrees of hindgut rotation and extension simultaneously in a live embryo, we developed a novel procedure. The hindgut has a hook-like structure, and the peak of the elbow-shaped bend in this structure was defined to evaluate hindgut rotation (Fig 2A). The root of the hook-like structure is largely straight; therefore, the direction of the hindgut lumen is parallel to its rotation axis, which was defined as the pivot line (S1 Fig). Thus, as the hindgut rotates, the distance between the peak of the elbow-shaped bend and the pivot line increases (S1 Fig). The distance between the pivot line and the center of the hindgut lumen at the peak of the elbow-shaped bend was measured at 0 and 60 min (Fig 2A). The change in these values from 0 to 60 min was referred to as the rotational movement index. Moreover, to quantify hindgut elongation, a line passing through the most anterior peak of the hook-like structure and perpendicular to the pivot line was drawn. The distance between the line and the posterior end of the curved part in the hook-like structure was measured at 0 and 60 min (Figs 2C and S1). The ratio of the values at 60 min to those at 0 min was defined as the elongation rate.

Using this novel procedure, the rotation and elongation of the hindgut were compared among wild-type embryos, *E-cad*-mutant embryos, and *Myo1D-*overexpressing embryos in the hindgut epithelium. In the *E-cad* mutant, the rotational movement index was almost negligible. Moreover, the reduction of the value was significant compared with that in the wild type, which coincided with the elimination of cell sliding under this condition, as described above (Fig 2B). However, in these embryos, the elongation rate was largely comparable to that in the wild type (Fig 2D). Therefore, no correlation was noted between hindgut rotation and elongation. These values were also assessed in the hindgut of *Myo1D-*overexpressing embryos. Under this condition, the rotational movement index was significantly higher than that in the wild type, which coincided with the increase in cell sliding, as described above (Fig 2B). In contrast, the elongation rate did not differ markedly between the wild-type and *Myo1D*-overexpressing embryos, demonstrating the discordance between these two events (Fig 2D). These results suggest that *E-cad* and *Myo1D* affect cell sliding independently of convergent extension.

### *E-cad* and *Myo1D* affected cell sliding independently of cell intercalation

Convergent extension is driven by cell intercalation, which is observed as a type of anisotropic junction remodeling, previously designated as T3 to T1 transition (Fig 2E) [4]. This transition drives these epithelial cells to intercalate with each other, resulting in the elongation of the hindgut epithelial tube [10]. Junctional remodeling has been considered unrelated to cell sliding or hindgut rotation because most cell boundaries were maintained during cell sliding, and the cell boundaries diminished by junctional remodeling did not exhibit LR bias [22]. Additionally, a simulation without junctional remodeling successfully recapitulated hindgut rotation [22]. Based on our aforementioned results, we speculated that cell intercalation is not affected in *E-cad*-mutant embryos or *Myo1D-*overexpressing embryos. To test this prediction, we examined the frequencies of the cell intercalation in the hindgut epithelium of these embryos. In the wild type, the cell intercalation was detected in 9.3% of examined cells in 0–60-min intervals, as reported previously (Table 1) [22]. In the *E-cad* mutant, in which cell sliding was largely abolished, the cell intercalation was still observed at a frequency of 12.7% in the same interval, supporting the independent regulation of these two cellular dynamics (*shg*^*69B*^ in Table 1 and Fig 2F). Furthermore, in the embryos overexpressing *Myo1D* in their hindgut, the cell intercalation was observed at a frequency of 7.8% in the same interval, which was largely comparable with that in the wild type (Table 1 and Fig 2G). These results suggest that the deceleration and acceleration of cell sliding do not affect the frequency of cell intercalation.

**Table 1:** The frequency of cell intercalation.

genotype	intercalation index	n	N
Wild type	9.3%	151	5
*shg* ^*69B*^	12.7%	173	7
*byn-gal4*, *UAS-Myosin1D*	7.8%	192	5
*baz* ^*4*^	2.0%	255	7

n and N represent cell and embryo numbers, respectively.

As the T3 to T1 transition may exclusively contribute to hindgut elongation, we predicted that this anisotropic junction remodeling should not exhibit an LR bias. Previously, we analyzed the angle θ between the diminishing cell boundaries and the vertical line to the pivot line in the wild type and reported that cell boundaries with an angle θ less than 10 degrees were selectively diminished, although the angle θ of diminishing cell boundaries did not exhibit a detectable LR bias in the wild type [22]. In this study, we analyzed the angle θ of diminishing cell boundaries in *E-cad*-mutant embryos and *Myo1D*-overexpressing embryos. Although cell sliding activity significantly differed in these embryos (Fig 1H), we did not observe a marked difference in the angle distribution of diminishing cell boundaries (Fig 2H and 2I). Furthermore, we did not detect an LR bias at these frequencies under these two conditions (Fig 2H and 2I). These results further suggest that cell sliding is not coupled with cell intercalation.

Although we did not observe marked changes in cell junction remodeling upon the modulation of cell sliding, we revealed that the chiral dynamics of cell junctions, detected as cell boundary rotation, were coupled with cell sliding but not with junction remodeling. As described above, we measured the angle θ of cell boundaries at 0 and 60 min and the rotational angle of each cell boundary from 0 to 60 min [22]. Then, we assessed the frequency of cell boundaries (%) that showed counterclockwise or clockwise rotation (Fig 2J and 2K) [22]. Compared with the wild type, we found that the LR bias of cell boundary rotation largely disappeared in the *E-cad* mutant (Fig 2K). In contrast, compared with the wild type, the LR bias of cell boundary rotation was augmented in *Myo1D-*overexpressing embryos (Fig 2K). Therefore, cell sliding correlated with cell boundary rotation but not with cell junction remodeling under these two genetic conditions. These results further suggest that cell sliding and cell intercalation are distinct epithelial dynamics and that *E-cad* and *Myo1D* preferentially contribute to the former.

### Par-3/Bazooka (Baz) affected convergent extension but not cell sliding

**Fig 3:**
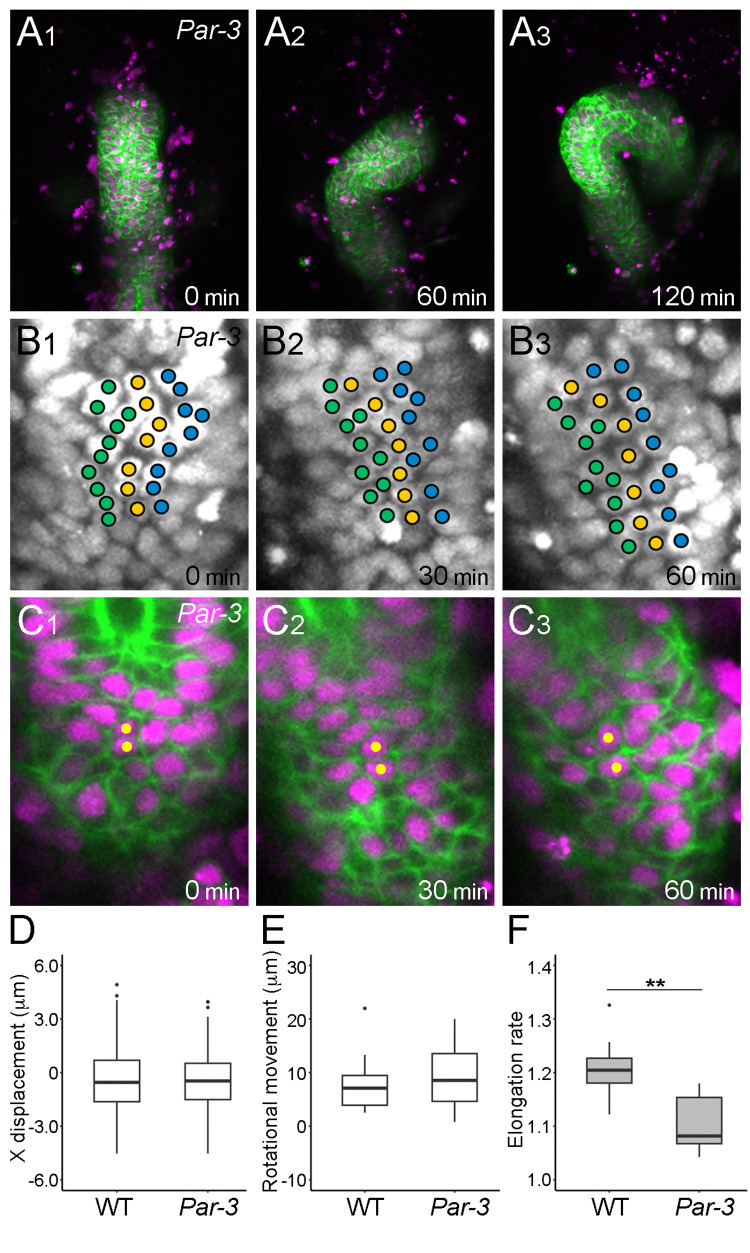
*Par-3* mutation impaired hindgut elongation. (A1–A3) Still shots from a time-lapse Video of hindgut rotation in the *Par-3* homozygote visualized as described in Fig 1A. (B1–B3) Displacement of nuclei in the hindgut epithelium of the *Par-3* homozygote visualized as described in Fig 1D. (C1–C3) Still shots from a time-lapse Video of cell nuclei (magenta) and cell boundaries (green) in the *Par-3*-homozygote. Yellow circles indicate cells next to each other from 0 to 60 min. (D–F) Quantification of *Par-3*-mutant phenotypes. (D) Boxplots of x displacement in the wild type (WT) (n = 164, N = 7) and *Par-3* homozygote (n = 296, N = 9). (E, F) Boxplots of rotational movement index (μm) (E) and elongation rate (F) in the WT (N = 9) and *Par-3* homozygote (N = 7). In A1–C3, the anterior is on top, and the time elapsed from the start of the Video is shown on the lower right. In D–F, black dots are outliers. **p < 0.01.

We next analyzed whether genes affecting convergent extension also play roles in cell sliding. Lengyel *et al*. identified various mutants affecting the convergent extension of the *Drosophila* hindgut [17,18]. However, as these mutants, including *bowl*, *lines*, and *drumstick* mutants, showed severe defects in hindgut development, it was difficult to properly evaluate the rotation and elongation of the hindgut using our procedure (S2A–S2C Fig). In a recent study, a mutant of *Par-3*/*baz*, encoding *Drosophila* Par-3, showed defects in convergent extension during germband elongation [32]. In this study, we found that embryos homozygous for a classical allele of *baz*, *baz*^*4*^, a presumptive null mutant, showed elongation defects in the hindgut (Fig 3A and S7 Video) [33,34]. Approximately 25% of these embryos had obscure hindgut morphology (designated as deformation), which led us to exclude them from analyzing LR asymmetry (S2D Fig). However, in the remaining embryos (approximately 75%), the hindgut had a hook-like structure. Most of them showed normal dextral LR asymmetry, based on the curving direction, although they occasionally showed delayed germband retraction, as reported previously (S2D and S2E Fig). In the hindgut of these embryos, the x displacement (cell sliding) and rotational movement index were equivalent to those in the wild type, demonstrating normal hindgut rotation (Fig 3B, 3D and 3E and S8 Video). However, their elongation rate was significantly reduced in the *Par-3*/*baz* mutant (Fig 3C), which coincided with a severe decrease in the cell intercalation to a frequency of 2.0% compared with 9.3% in the wild type (Table 1 and Fig 3F). However, the observed reduction in hindgut elongation detected in our assay could be explained if the hindgut leaned ventrally. To exclude this possibility, we measured the depth of the hindgut along the dorsal-ventral axis (DV depth) at 0 and 60 min (S3A–S3D Fig). Our results demonstrated that DV depth was similar between wild type and *Par-3* mutant hindgut, and the ratios at 0–60 min were equivalent for both genotypes (S3A, S3C and S3D Fig). Conversely, the DV depth of *E-cad* mutant differed from that of the wild type due to suppressed hindgut rotation (S3B Fig). These findings indicate that wild-type Par-3 regulates hindgut elongation through cell intercalation without affecting cell sliding and hindgut rotation. Based on these results, we speculate that cell sliding and cell intercalation are concurrently executed via two distinct and independent mechanisms in the hindgut epithelium.

### Computer model recapitulated that cell sliding and convergent extension are independently but concurrently executed through contractile force with distinct polarities

**Fig 4:**
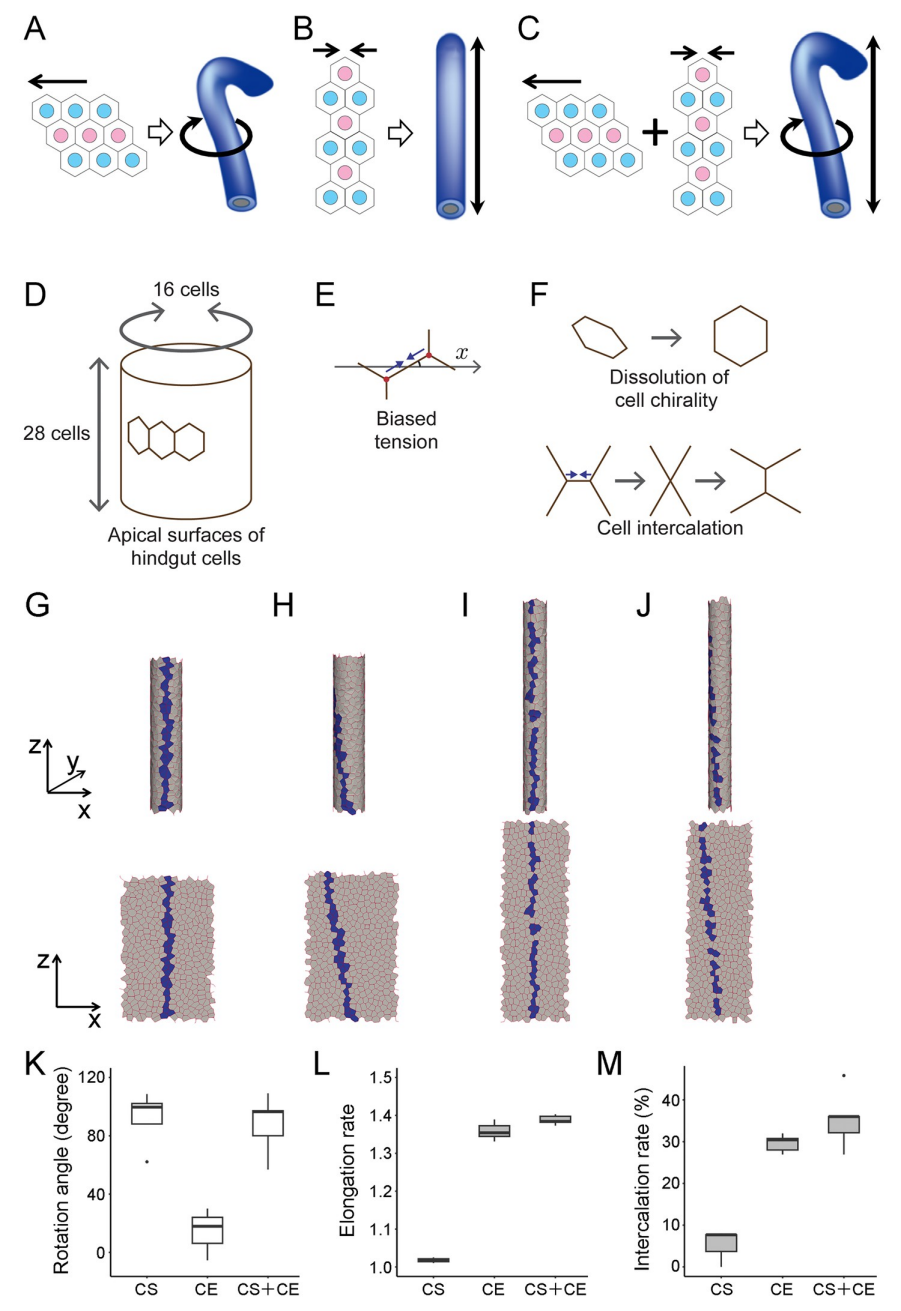
Simulations verified that cell sliding and cell intercalation are independently executed. (A–C) Schemas showing that cell sliding (A) and cell intercalation (B) regulate hindgut rotation and elongation, respectively, and both together cause hindgut rotation and elongation simultaneously (C). (D, E) Schemas of the vertex dynamics model. (D) The model tube represents the apical surfaces of the hindgut epithelial cells, which are approximately 16 cells in circle and 28 cells in length. (E) Biased contraction, dependent on edge angle was introduced (indicated by two arrows). (F) To recapitulate cell sliding, initial cell chirality was formed by rightward-tilted-edge biased contraction and its subsequent dissolution were introduced (upper). To recapitulate cell intercalation, horizontally biased edge contraction (two arrows) was introduced, which occasionally resulted in the T3 to T1 transition of the edges (lower). (G–J) Simulations of gut deformation. Initial condition with cell chirality (G), and final shapes (H–J) of the model tube in 3D (upper) and 2D (lower) resulting from simulations with cell sliding only (H), cell intercalation only (I), and both simultaneously (J). Coordinate axes in 3D (*x*, *y*, and *z*; upper) and 2D (*x* and *y*; lower) are shown on the extreme left. Model cells forming a line at the front face in the initial phase (G) are labeled in blue and are chased until the end (H, I, J). (K, L, M) Quantification of rotation angle, elongation, and intercalation rates was performed *in silico* from 5 independent simulations for each condition. (K) Boxplots of rotation angle (degree). (L) Boxplots of elongation rate. (M) Boxplots of intercalation rate (%). Black dots are outliers. CS, model implementing only cell sliding; CE, model implementing only convergent extension; CS + CE, model implementing both cell sliding and convergent extension.

Our analyses demonstrated that cell sliding and convergent extension are independently but concurrently regulated in the hindgut epithelium. To understand the underlying mechanisms by which these two epithelial dynamics are integrated, we performed numerical simulations using vertex dynamics models [35–38]. Previous studies have shown that cell sliding and convergent extension rely on two distinct contractions of cell boundaries; contractions with chirality and anterior–posterior/distal–proximal anisotropy are responsible for cell sliding and convergent extension, respectively [4,22]. Therefore, we hypothesized that these two contractions account for the rotation and extension of the hindgut, which occur independently of each other but simultaneously (Fig 4A–4C).

Our previous studies showed that introducing cell chirality by contracting rightward-tilted cell edges and subsequently dissolving this chirality can induce cell sliding, resulting in hindgut rotation [9,22]. In this study, we enhanced this model by incorporating the axial and radial expansion and contraction of the hindgut, which facilitates hindgut elongation (refer to Eq 1 in Materials and Methods). In brief, a regular cylinder with 448 regular hexagons (16 hexagons in circle and 28 hexagons in length; the area is 1) was created as a model tube representing the apical (luminal) surfaces corresponding to the straight region of the hindgut (Fig 4D). Anisotropic contraction of the cell boundaries was introduced as edge tension biased at oriented angles (Fig 4E). Cell chirality was represented by initial cell shape strain (Fig 4F top left, and 4G), induced by biased edge tension maximal at 45 degrees from the *x*-axis. As the simulation progressed, this tension was dissolved (Fig 4F top right) and a horizontal biased edge tension, maximal at 0 degrees was added (Fig 4F bottom).

In our current model, cell boundary contractions with chirality or anterior–posterior/distal–proximal anisotropy were separately introduced. The introduction and subsequent dissolution of chiral tension caused the model tube to rotate approximately 90 degrees counterclockwise, as viewed from the bottom of the tube (Fig 4G and 4H). Additionally, applying edge tension with anterior–posterior/distal–proximal anisotropy resulted in axial elongation of the model tube (Figs 4I and S4). Notably, integrating both tensions led to simultaneous tube rotation and elongation, effectively recapitulating hindgut morphogenesis *in vivo* (Figs 4J and S4).

The deformations of model tubes were quantified by averaging the results of five independent simulations for each condition. The incorporation of cell chirality with or without the anterior–posterior/distal–proximal anisotropic tension induced approximately 90 degrees of rotation, whereas the anterior–posterior/distal–proximal anisotropic tension alone induced almost no rotation (Fig 4K). Moreover, the anterior–posterior/distal–proximal anisotropic tension induced approximately 1.4-fold axial elongation with or without the incorporation of cell chirality, whereas cell chirality alone did not induce any axial elongation (Fig 4L). As the wild-type hindgut tube exhibited approximately 1.2-fold elongation for 1 h (Fig 2D) and 90-degree rotation for 2 h (Fig 1A) *in vivo*, our *in silico* models quantitatively agreed with the *in vivo* behaviors of the hindgut tube. In the wild-type hindgut, cell intercalation occurred at 9.3% of the cell boundaries every 30 min (Table 1). Consistent with these observations, the wild-type model exhibited an average cell intercalation rate of 35.4% over a rotation and elongation period of approximately 2 h *in vivo* (Fig 4M). These results further supported our hypothesis that contraction forces with distinct polarities cause two independent tissue deformations, which are integrated by occurring in the same developmental window.

### MyoII contributed to both cell sliding and cell intercalation

Our *in silico* models predicted that contraction forces with distinct polarities, namely chirality and anterior–posterior/distal–proximal anisotropy, are crucial for the rotation and elongation of the hindgut. Although it is difficult to specifically inhibit only one of these two contraction forces, it is known that MyoII regulates the contractile force of cell boundaries in various epithelial dynamics [4,6,39]. Therefore, the validity of our *in silico* model could be evaluated by examining whether the disruption of MyoII-dependent contraction forces in the hindgut and in the *in silico* models has comparable consequences.

In *Drosophila*, the MyoII heavy chain (MHC) is encoded by *zipper* (*zip*). We performed live imaging of the embryonic hindgut homozygous for *zip*^*2*^, a null allele of *zip* [40]. However, the *zip*^*2*^-homozygous embryo showed normal hindgut rotation and elongation, probably because the maternal supply of *zip* is sufficient for supporting the normal morphogenesis of the hindgut (S3A Fig) [41]. Hence, to further inhibit *MyoII*, we misexpressed a dominant-negative form of *zip*, *DN-zip*, in the hindgut epithelium of *zip*^*2*^-heterozygous embryos [42]. We noted LR inversion and bilateral phenotypes of the hindgut, suggesting that MHC is required for proper hindgut rotation (S3B and S3C Fig). We also examined a null mutant of *spaghetti squash* (*sqh*), which encodes the myosin regulatory light chain (MRLC) [43]. In embryos homozygous for *sqh*^*AX3*^, a null allele of *sqh* [44], we noted LR inversion and no laterality phenotypes of the hindgut, suggesting that MRLC is also important for hindgut rotation (S3B Fig). However, the percentages of LR defects in these mutants were too low to conduct quantitative analyses at a cellular level, probably because of the residual activity of MyoII.

**Fig 5:**
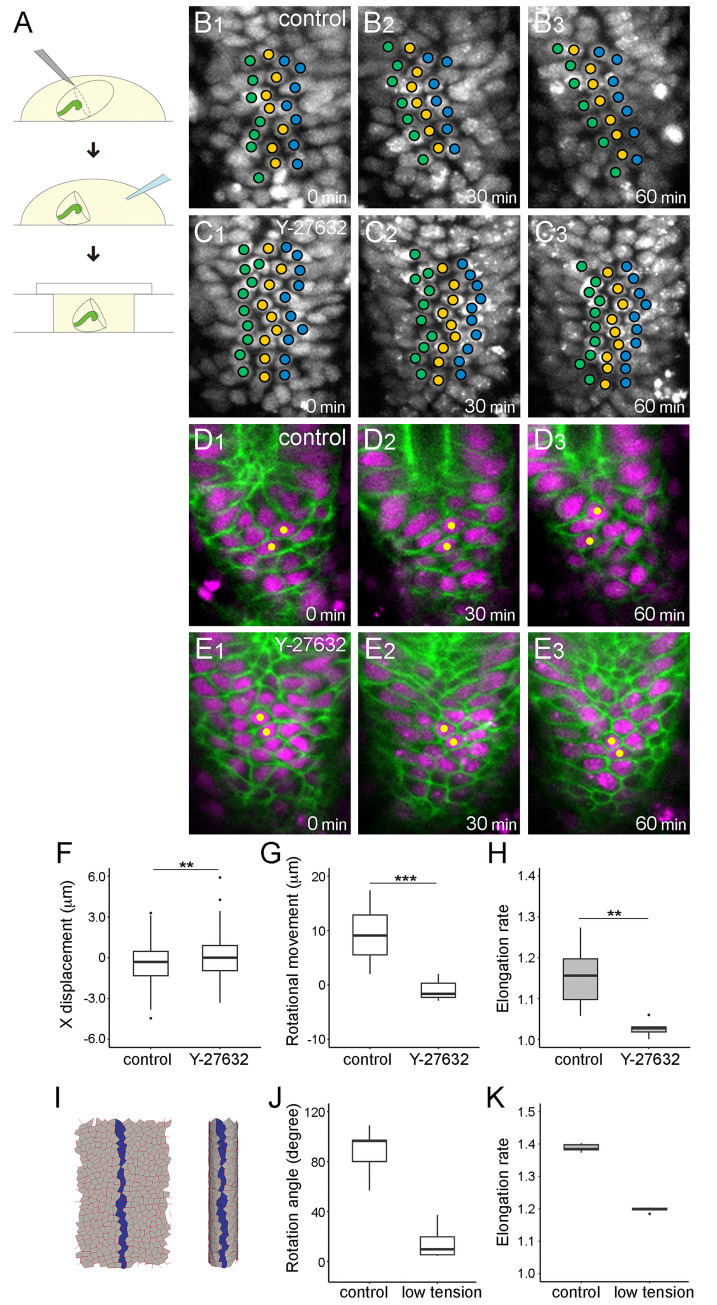
Inhibition of Myo II activity stopped the rotation and elongation of the hindgut. (A) Schema of drug treatment *ex vivo*. Late-stage 12 embryos were dissected using a glass pipette needle (top), treated with a drug or DMSO (control) (middle), and encapsulated with a coverslip to perform time-lapse imaging (bottom). (B1–C3) Displacement of nuclei in the wild-type hindgut epithelium cultured *ex vivo*, visualized as described in Fig 1D. The cultured hindgut was treated with DMSO (B1–B3, control) or an inhibitor against MyoII, Y-27632 (C1–C3, Y-27632). (D1–E3) Still shots from a time-lapse Video of cell nuclei (magenta) and boundaries (green) in the hindgut cultured *ex vivo*, visualized as described in Fig 1A. The cultured hindgut was treated with DMSO (D1–D3, control) or an inhibitor against MyoII, Y-27632 (E1–E3, Y-27632). In B1–E3, the anterior is on top, and the time elapsed from the start of the Video is shown on the lower right. (F) Boxplots of x displacement in the wild-type hindgut cultured *ex vivo* with DMSO (control) (n = 155, N = 5) or with Y-27632 (Y-27632) (n = 232, N = 5). (G, H) Boxplots of rotational movement index (μm) (G) and elongation rate (H) in the wild-type hindgut cultured *ex vivo* with DMSO (control) (G, N = 8; H, N = 7) or with Y-27632 (Y-27632) (G, N = 9; H, N = 8). (I–K) Results of simulation in which edge tension was reduced after the initial introduction of chirality. (I) Final shape of the model tube in 3D (right) and 2D (left). Model cells forming a line at the front face in the initial phase are labeled in blue and are chased until the end. (J, K) Boxplots of rotation angle (degree) (J) and extension rate (K) in models implementing both cell sliding and convergent extension with normal (control) or reduced (low tension) edge tensions. In F, H, and K, black dots are outliers. ***p < 0.001. **p < 0.01.

To solve this problem, we developed an *ex vivo* assay, which was derived from an *ex vivo* organ culture system reported previously [22]. In brief, we dissected the most posterior part of the embryo, which is mainly composed of the hindgut, before the initiation of its rotation [22]. In this new system, we treated the cultured hindgut with chemical inhibitors and quantitatively analyzed its morphological changes *ex vivo* (Fig 5). We found that the hindgut rotated and elongated *ex vivo*, as observed *in vivo* (S9 Video). Using this *ex vivo* system, we administered an inhibitor against MyoII, Y-27632, which blocks the MyoII activator Rho kinase, into the cultured hindgut (S10 Video) [45]. We assessed the rotational movement index *ex vivo* under this condition using the same procedures as those used *in vivo*. The rotational movement index was almost zero in the wild-type hindgut treated with Y-27632 *ex vivo*. However, the rotational movement index in the mock-treated hindgut (control) was equivalent to that in the wild type *in vivo* (Fig 5G). Therefore, hindgut rotation was diminished by the suppression of MyoII activity. We then analyzed the effect of Y-27632 treatment on cell sliding. In the mock-treated hindgut cultured *ex vivo*, the columns of nuclei lined up along the anterior–posterior axis at 0 min slanted to the left after 30 and 60 min, as noted in the wild type *in vivo* (Fig 1D1–1D3 and 5B1–3, S11 Video). However, the columns of nuclei in the wild-type hindgut treated with Y-27632 *ex vivo* did not change their direction for 60 min, demonstrating that cell sliding was abolished (Fig 5C1–5C3 and S12 Video). This result was quantitatively supported by our findings that x displacement was negligible in the wild-type hindgut treated with Y-27632 *ex vivo*, whereas x displacement in the mock-treated hindgut *ex vivo* was largely equivalent to that in the wild type *in vivo* (Fig 5F). These results suggest that the activity of MyoII is essential for cell sliding.

Using the same *ex vivo* assay, we assessed the requirement of MyoII for hindgut elongation. We found that the elongation rate was close to one (no elongation) in the wild-type hindgut treated with Y-27632 *ex vivo*. However, the mock-treated hindgut elongated to a similar extent as the wild type *in vivo* (Fig 5H). These results suggest that the activity of MyoII is also essential for the convergent extension of the hindgut. Therefore, we predicted that T3 to T1 cell junction remodeling, which drives convergent extension, is impaired under this condition. We found that the T3 to T1 transition (cell intercalation) was severely inhibited by the addition of Y-27632 compared with that in the mock-treated wild type *ex vivo* (Table 2, Fig 5D and 5E). Collectively, our results suggest that MyoII-dependent activity, which induces contraction force along cell boundaries, is required for both cell sliding and convergent extension.

**Table 2:** The frequency of cell intercalation in *ex vivo* experiments.

condition	intercalation index	n	N
*ex vivo* control	5.8%	155	5
*ex vivo* Y-27632 treated	0.4%	232	6

n and N represent cell and embryo numbers, respectively.

We next validated these results in our *in silico* model. We concurrently diminished the dissolution of cell chirality and contractions of cell boundaries with anterior–posterior/distal–proximal anisotropy by reducing edge tensions in the model, although chirality was introduced before edge tension reduction (Fig 5I). We found that this model gut showed decreases in rotation and elongation (Fig 5I–5K), which is similar to the findings in the hindgut in which MyoII activity was inhibited *ex vivo* (Fig 5C, 5E, 5G and 5H). Thus, our *in silico* model qualitatively recapitulated the situations. Taken together, our results suggest that cell sliding and convergent extension are distinct epithelial dynamics induced by chiral and anterior–posterior/distal–proximal anisotropic contractions, which operate parallelly in the hindgut epithelium in the same developmental window.

## Discussion

Epithelial morphogenesis often involves multiple types of tissue deformations that occur concurrently^2^. For example, in *Drosophila*, the late stage of gastrulation is coupled with the convergent extension of germband elongation. Some pioneer studies revealed that gastrulation and germband elongation are separable events. This finding facilitated the analyses of specific machineries responsible for each of these events [4,16,39]. However, in general, it is still difficult to study these morphogenetic events that occur simultaneously in the epithelium because we need to find proper objects that will enable the specific detection of the effects of experimental modulations of each event. Therefore, we developed a procedure to analyze two distinct events simultaneously occurring in the hindgut epithelium: cell sliding and convergent extension [10,22].

Our analysis revealed that cell sliding in the hindgut epithelial tube depends on *Myo1D* and *E-cad*. However, we failed to detect the requirement of these two genes for convergent extension, which is revealed by the T3 to T1 transition that simultaneously occurs with cell sliding. On the other hand, convergent extension depends on *Par-3*, which is not required for cell sliding. These results suggest that cell sliding and convergent extension are accomplished by distinct cascades, although they function concurrently in the same epithelium. MyoII is simultaneously required for both cell sliding and convergent extension of this tissue. Therefore, although cell sliding and convergent extension depend on different cascades, they share MyoII as a key factor, presumably through the control of contraction forces, as observed in various epithelial deformations [4,39,46–49].

These ideas were verified through computer simulations. Our vertex model with the parameters for cell boundary contractions with chirality and anterior–posterior/distal–proximal anisotropy successfully recapitulated the hindgut deformations observed *in vivo*: cell sliding and convergent extension. Importantly, a model executing cell boundary contractions with chirality but not anterior–posterior/distal–proximal anisotropy demonstrated cell sliding without convergent extension, which is similar to the findings in the hindgut of the *Par-3*/*baz* mutant. On the other hand, a model executing cell boundary contractions with anterior–posterior/distal–proximal anisotropy but not chirality demonstrated convergent extension without cell sliding, which is similar to the findings in the hindgut of the *E-cad* mutant. Thus, the modifications of parameters in our simulation coincide with the consequences of genetic alterations *in vivo*. Furthermore, when edge tension was reduced in the steps of cell chirality dissolution and anterior–posterior/distal–proximal anisotropic cell boundary contraction in our model, cell sliding and convergent extension decreased together; this result is in accordance with the conditions of the hindgut in which MyoII activity was inhibited (Fig 5C and 5E–5H). As the activities of MyoII generally contribute to the generation of tension along cell boundaries, this agreement also supports the validity of our model (Fig 5I–5K). These results indicate that cell sliding and convergent extension are governed by distinct mechanical processes that depend on two different genetic pathways, both of which involve tension along cell boundaries induced by MyoII.

As both cell sliding and convergent extension depend on MyoII, biochemical cascades controlling MyoII, which function parallelly in epithelial tissues, could be complex and delicate. However, based on our current understanding of how MyoII regulates a broad range of mechanical properties in a cell, such biochemical complexity may not be overwhelming. For instance, it is known that many specific functions of MyoII are specially regulated through its confined localization in an epithelial cell, e.g., the cell cortex, apical cell boundaries, and tricellular junctions [4,39,50,51]. Furthermore, specific activities of MyoII are regulated by phosphorylation on specific sites of MyoII [44,52]. Therefore, MyoII could simultaneously play different roles through such multiple layers of regulatory mechanisms. However, as reported previously, the subcellular distribution of MyoII did not show marked chirality in hindgut epithelial cells [22]. Therefore, how MyoII contributes to cell sliding remains unclear. However, we found that MyoII and Par-3/Baz are required for convergent extension through the promotion of the T3 to T1 transition (Figs 3C and 5E). Par-3/Baz is required for the preferential contraction of anterior and posterior borders in intercalating cells of *Drosophila* embryos [32]. Such preferential contraction is achieved by the planar polarized distribution of MyoII to the anterior and posterior borders of these cells [32]. However, we failed to detect the planar polarized distribution of MyoII in the hindgut epithelium (S3D and S3E Fig). Therefore, the underlying mechanisms controlling the local activity of MyoII may differ between the convergent extension of the hindgut and embryos. As we found that hindgut deformation can be resolved into at least two independent tissue dynamics, specific cellular events in each of them can be separately studied in the future, making it easier to understand their molecular mechanisms.

Cell intercalation associated with LR asymmetric morphogenesis has been reported in the development of the mouse and Xenopus stomachs, and the zebrafish heart [53,54]. In mouse and *Xenopus* stomachs, radial cell rearrangement expands the left side wall more than the right, causing the stomach tube to bend leftward as if rotating [53]. In the zebrafish heart tube, cells on the left intercalate more actively than those on the right, thereby driving the clockwise rotation of the heart tube [54]. In these cases, cell intercalation triggers LR asymmetric morphogenesis. However, in the *Drosophila* embryonic hindgut, cell intercalation does not affect LR asymmetry but instead causes tissue elongation. Therefore, this indicates that cell intercalation in LR asymmetric development is evolutionarily divergent. Additionally, tissue elongation in the anterior-posterior (cranial-caudal) direction may enhance LR asymmetric morphogenesis in confined body cavities. In chick embryos, gut tube elongation following LR asymmetry determination by the dorsal mesentery induces LR asymmetric looping of the organ [1,55]. During heart tube development in chick embryos, tissue elongation enhances the looping structure [1]. However, as reported in this study, in the *Drosophila* embryonic hindgut of the *Par-3* mutant in which tissue elongation was absent, the extent of LR asymmetry in this organ was comparable to that in the wild type, indicating that tissue elongation does not enhance LR asymmetry. Therefore, the independent regulation of LR asymmetric rotation and anterior-posterior elongation in the *Drosophila* hindgut suggests that divergent mechanisms exist in morphogenesis associated with LR asymmetry formation of internal organs among species.

## Materials and methods

### Fly strains

The following fly strains were used: *shg*^*R69*^ [27], *zip*^*2*^ (BL8739) [40], *sqh*^*AX3*^ (BL25712) [44], *baz*^*4*^ (BL3295) [33,34], *byn*-gal4 [56], NP2432-gal4 (Kyoto 104201), UAS-*myrGFP-p10* (JFRC29) [30], UAS-*RedStinger* (BL8547), UAS-*Myo31DF* [25], UAS-*DN-zip* [42], and UAS-*sqh-GFP* [22]. They were raised at 18°C or 25°C. The genotypes used for live imaging of *shg* homozygotes were *shg*^*R69*^/*shg*^*R69*^, *NP2432*; UAS-*RedStinger*/UAS-*myrGFP-p10* and those used for live imaging of *baz* homozygotes were *baz*^*4*^/*baz*^*4*^; UAS-*RedStinger*/*byn*-gal4, UAS-*myrGFP-p10*. For wild-type embryos overexpressing *Myo31DF*, the genotypes UAS-*Myo31DF*/+; UAS-*RedStinger*/*byn*-gal4, UAS-*myrGFP-p10* were used to record time-lapse Videos. For the selection of *shg*^*R69*^ and *zip*^*2*^ mutants, *Cyo-sChFP* (BL35523) and *Cyo-GFP* (BL5195) balancers were used. The *FM7c-GFP* (BL5193) balancer was used for selecting the *baz*^*4*^ mutant.

### Live imaging and quantification of parameters for characterizing hindgut deformation

Live imaging of the hindgut epithelium was performed as described previously [22]. Late-stage 12 embryos of the appropriate genotype were mounted with the dorsal side on top in oxygen-permeable halocarbon oil 27 (Sigma-Aldrich) using 0.17–0.25-mm-thick coverslips (thickness no. 2, Matsunami Glass) as spacers. Time-lapse Videos were recorded every 5 min for 2 h using a scanning laser confocal microscope (LSM700, Zeiss) at 23°C–25°C.

For quantifying cell displacement in these time-lapse Videos, centers of nuclei detected by the expression of UAS-*RedStinger* were manually defined. The root part of the hindgut, the region 20%–40% away from the bottom, and the central three columns of cells were analyzed to avoid extraneous effects of tissue movement (Fig 1D) [22]. To track the movement of nuclei, the depth of the z stack was adjusted at each time point to make the time-lapse Videos clearer because these nuclei changed their depth from the object glass over time. The *x*- and *y*-coordinates of nuclei in these time-lapse Videos were measured every 30 min until 60 min after the beginning of hindgut rotation using ImageJ software (https://imagej.net/ij/). The position of the subjacent nucleus was set at (0, 0) coordinates, and the relative displacement of the upper nucleus in the x direction was quantified every 30 min until 60 min after the beginning of hindgut rotation (Fig 1G). Welch’s t-test was used for statistical analysis.

To analyze the rotation of cell boundaries detected by the expression of UAS-*myrGFP-p10* in the hindgut epithelium, the angle changes in the boundaries between cells aligned along the central column were measured every 30 min until 60 min after the beginning of hindgut rotation. The changes in these angles were then classified into counterclockwise, unchanged, and clockwise (Fig 2J), and the frequencies of each class were calculated. Statistical analysis was performed using χ^2^ test.

To quantify the frequency of cell intercalation in hindgut epithelial cells, cell boundaries that were separated through the intercalation of other cells were counted every 30 min until 60 min after the beginning of hindgut rotation. This number was divided by the total number of examined cells, which was designated as the intercalation index. Moreover, the angle of cell boundaries diminished by cell intercalation against the anterior–posterior axis of the hindgut was measured, and the percentage of these cell boundaries with every 20-degree angle bin was calculated.

Hindgut rotation was quantitatively assessed as an increase in the distance between the peak of the elbow-shaped bend and the pivot line from 0 to 60 min, which was designated as the rotational movement index (S1 Fig). To quantify hindgut elongation, a line passing through the most anterior peak of the hook-like structure and perpendicular to the pivot line was drawn. The distance between the line and the posterior end of the hook-like structure was measured at 0 and 60 min. The ratio of the distance at 60 min to that at 0 min was designated as the elongation rate (Figs 2C and S1). The DV depth was measured as the distance between the most dorsal part of the gut lumen and the most anterior peak of the hook-like structure along the z (dorsal-ventral-axis) direction at 0 and 60 min (S1 and S3A–S3D Figs). Mann–Whitney U-test was used for statistical analysis.

### *ex vivo* culture and drug treatment

The hindgut with the most posterior part of the embryo was dissected in M3 medium (S8398, Sigma-Aldrich) with 10% fetal bovine serum (FBS, Biowest) and 1% trehalose (34413–92, Nakarai) on double sticky tape placed on a glass slide. The dissected hindgut was covered with a coverslip with 0.17–0.25-mm-thick spacers and continuously cultured for 2 h at 23°C–25°C under a microscope (LSM700). To inhibit MyoII in the culture, Y-27632 (Y0503, Sigma-Aldrich), an inhibitor against MyoII, dissolved in water was added to the culture medium at a final concentration of 100 μM after the dissection. Time-lapse Videos were recorded and analyzed as described above.

### Two-dimensional (2D) vertex model for hindgut extension and torsion

To analyze the effects of polarized contractions of cell boundaries, such as anterior–posterior/distal–proximal anisotropy and chirality, on hindgut deformation, we performed numerical simulations of epithelial torsion by enhancing a 2D vertex model.

In our model, the hindgut epithelium was assumed to be a deformable cylinder with variable perimeter and axial length represented by *L*_*x*_ and *L*_*y*_, respectively. The multicellular dynamics within the cylinder were calculated by expanding them to those in a rectangular sheet of width *L*_*x*_ and length *L*_*y*_ in the *xy*-coordinates, where the radial and longitudinal axes of the cylinder correspond to the *x-* and *y-*axes of the sheet, respectively. Periodic boundary conditions were applied at boundaries *x* = *L*_*x*_ and *y* = *L*_*y*_. To represent the longitudinal extension of the cylinder, the perimeter *L*_*x*_ and length *L*_*y*_ of the cylinder can vary depending on the tissue stress. Additionally, to represent the torsion of the cylinder, the *x*-coordinate can be shifted at the boundary from *y* = *L*_*y*_ to *y* = 0 with length *L*_*yx*_ according to the tissue stress.

The rectangular sheet forming the cylinder is densely lined with numerous cells. Each epithelial junction between neighboring cells was represented by an edge, and each cell shape was represented by a polygon containing the edges. The edges and vertices were shared with neighboring cells. Cell dynamics were calculated by tracking the motion of vertices, where the location vector of the *i*th vertex is represented by ***r***_*i*_. In addition, cell rearrangement was represented by reconnecting the edges when the length of the edges fell below a threshold value Δ*l*, based on the motion of vertices. The properties of all cells were assumed to be the same, and cell growth and division were not considered [7]. For simplicity, we ignored the effects of epithelial thickness.

To combine cylindrical deformation with multicellular dynamics, we followed a sequential calculation process: first, we calculated the geometry of cells ***r***_*i*_, from which we obtained the stress tensor of the entire tissue, represented by *σ*_*αβ*_. Using this stress tensor, we then calculated the geometry of the cylinder *L*_*x*_ and *L*_*y*_. The iterative calculations of these processes allowed us to calculate the combined dynamics of the cylinder and cells.

### Motion equation of a deformable tube

Hindgut torsion occurs independently of surrounding tissues [22]. Additionally, abnormalities in chirality and torsion can be rescued by expressing Myosin1D in the posterior intestinal epithelium [9,25]. These findings suggest that hindgut torsion is driven autonomously by actomyosin-dependent junctional contraction of the hindgut epithelium. Therefore, we focused on the effects of junctional contractions, disregarding external forces such as luminal pressure and other forces from the hindgut environment.

To describe the cylindrical extension, the motion on the size of the tube, *L*_*x*_ and *L*_*y*_, was given by:

$$\xi \frac{\partial L_{x}}{\partial t}=-L_{x}\sigma_{xx},\xi \frac{\partial L_{y}}{\partial t}=-L_{y}\sigma_{yy}.$$ (Eq 1)

In Eq (2), *σ*_*αβ*_ is a stress tensor of the system. Moreover, to describe the cylindrical torsion, the motion on the shift length *L*_*yx*_ was given by:

$$(\frac{\xi }{3})\frac{\partial L_{yx}}{\partial t}=-L_{y}\sigma_{yx}.$$ (Eq 2)

The coefficient *ξ*/3 was given assuming that the hindgut is an isotropic material. Vertex positions were shifted in an affine manner according to the changes in *L*_*y*_.

### Motion equation of cells

The location vector of the *i*th vertex, represented by ***r***_*i*_, was given by:

$$\eta \frac{dr_{i}}{dt}=-\nabla U,$$ (Eq 3)

where *η* is a friction coefficient and *U* is a potential energy. The potential energy *U* was given by:

$$U\equiv \sum_{i}^{cell}\frac{K}{2}(1+\zeta {\left(1-\frac{A_{eq}}{A_{i}}\right)}^{2}){\left(A_{i}-A_{eq}\right)}^{2}+\sum_{i}^{cell}\frac{\Gamma }{2}{\left(P_{i}-P_{eq}\right)}^{2}+\sum_{j}^{edge}\Lambda_{j}L_{j}.$$ (Eq 4)

The first term describes the energy of area elasticity with the elastic coefficient *K*, for which *A*_*i*_ is the area of the *i*th cell and *A*_eq_ is the preferred area. The coefficient *ζ*(1−*A*_eq_/*A*_*i*_)^2^ in the first term regulates the individual cell size, preventing excessive shrinkage or expansion, where *ζ* represents the constraint strength. The second term describes the energy of perimeter elasticity with the elastic coefficient *Γ*, for which *P*_*i*_ is the perimeter of the *i*th cell and *P*_eq_ is the preferred perimeter. This term reflects the summation of the contractility of the actomyosin ring of individual cells and cell–cell adhesion at junctions between cells. The third term describes the line energy of junctions with active line tension, *Λ*_*j*_. This term represents both polarized contractions of cell boundaries, including anterior–posterior/distal–proximal anisotropy and chirality. The detailed implementation of these polarized contractions is described in the following simulation procedure. Here, *L*_*j*_ denotes the length of the *j*th edge. Line tensions can be changed by actin–myosin contractility. The line tension *Λ*_*j*_, depending on the angles of edges, was given by:

$$\Lambda_{j}={\Lambda_{c}+\Lambda }_{a}\cos\;2(\theta_{j}-\theta_{ref})+\Lambda_{r}w_{j},$$ (Eq 5)

where *θ*_*j*_ is the angle between the polar axis and the *j*th edge. The constant *θ*_ref_ is the angle of cell polarity that maximizes *Λ*_*j*_ (= *Λ*_*α*_) when *θ*_*j*_ = *θ*_ref_. The third term denotes the fluctuation force of actomyosin contractility with strength *Λ*_*r*_, where *w*_*α*_ satisfies 〈*w*_*α*_(*t*)〉 = 0 and $\langle w_{\alpha }(t_{1})w_{\beta }(t_{2})\rangle =\delta_{\alpha }\delta_{\beta }exp(-|t_{1}-t_{2}|/\tau_{R})$ $\langle \xi_{\beta }(t_{1})\xi_{\varrho }(t_{2})\rangle =\delta_{\beta }\delta_{\varrho }exp(-|t_{1}-t_{2}|/\tau )$, where *δ*_*α*_ is the Kronecker delta and *τ*_R_ is the correlation time of the noise as used previously in cell flow models [36,37].

### Stress tensor under periodic boundary condition

The stress tensor under the periodic boundary condition was given by:

$$\sigma_{\alpha \beta }\equiv \frac{1}{A_{i}}\sum_{i}^{cell}A_{i}\;\sigma_{\alpha \beta }^{i},$$ (Eq 6)

where *A*_*i*_ and $\sigma_{\alpha \beta }^{i}$ are the area and stress tensor of the *i*th cell, respectively. The stress tensor of the *i*th cell was given by:

$$\sigma_{\alpha \beta }^{i}\equiv \frac{1}{A_{i}}\sum_{j(i)}^{vertex}r_{\alpha }^{j(i)}f_{\beta }^{j(i)}.$$ (Eq 7)

Here, $r_{\alpha }^{j(i)}$ the *α*-component of the distance vector from the center of the *i*th cell. $f_{\alpha }^{j(i)}$ is the *α*-component of the force of the *j*th vertex. The potential energy *U* was divided into those of individual cells as follows:

$$U\equiv \sum_{i}^{cells}u_{i}$$ (Eq 8)

where the linear energy in the third term of Eq (4) is equally distributed between two adjacent cells. Assuming the quasistatic process, the force $f_{\alpha }^{j(i)}$ was given by:

$$f_{\alpha }^{j(i)}\equiv -\nabla u_{i}.$$ (Eq 9)

### Simulation procedure

Simulations were performed via two processes, i.e., precalculation (0≤*t*<*τ*_1_) and torsion (*τ*_1_≤*t*≤*τ*_2_). In both these processes, the boundary conditions of the *x-* and *y-*axes were set to periodic boundary conditions. During the precalculation, the angle of the polarized contraction of cell boundary was set to *θ*_ref_ = *π*/4 to represent the chirality causing chiral cell sliding. Additionally, during torsion, the angle was set to *θ*_ref_ = 0 to form the anterior–posterior/distal–proximal anisotropy of cell shapes, which drives convergent extension.

### Nondimensional values and parameter setting

$\sqrt{A_{eq}}$ was set to unit length, *KA*_eq_ to unit energy, and *η*/*K* to unit time. All the parameters used are listed in Table 3 [22,37,38]. For the simulation using reduced tension, *Γ* and *Λ*_*a*_ were reduced by 0.01 holds and *Λ*_r_ and *Λ*_r_ were reduced by 0.1 holds in torsion.

**Table 3:** Physical parameters.

Symbol	Unit	Values (pre)	Values (torsion)	Explanation
*A*_eq_	-	1	1	Unit
*P*_eq_		0	0	[38]
*K*	-	1	1	Unit
*η*	-	1	1	Unit
*ξ*	*η*	+∞	100	Much larger than *η*
*ζ*	-	0.01	0.01	-
*Γ*	*KA*_eq_	0.04	0.04	[38]
*Λ*_c_	*K*(*A*_eq_)^3/2^	0.12	0.12	[38]
*Λ*_*a*_	*K*(*A*_eq_)^3/2^	0.0075	0.014	Optimized
*θ*_ref_	-	*π*/4	0	Measured in experiments [22]
*Λ*_r_	*K*(*A*_eq_)^3/2^	0.03	0.015	Optimized
*τ*_R_	*η*/*K*	3.0	3.0	[37]
*N* (i.c.)	-	448	448	Measured in experiments (16 cells in circle and 28 cells in length)
***L***_***x***_ **(i.c.)**	$\sqrt{A_{eq}}$	9.92	-	Measured in experiments (length of the edge of a hexagon with A = 1 multiplied by 16 cells)
***L***_***y***_ **(i.c.)**	$\sqrt{A_{eq}}$	*NA*_eq_/*L*_*x*_	-	-

### Evaluation functions

To evaluate tube deformation, the normal strains of the system along the *x*- and *y*-axes, represented by *γ*_*xx*_ and *γ*_*yy*_, respectively, and the shear strain of the system, represented by *γ*_*yx*_, were given by:

$$\gamma_{xx}=\frac{L_{x}(\tau_{2})-L_{x}(\tau_{1})}{L_{x}(\tau_{1})},$$

$$\gamma_{yy}=\frac{L_{y}(\tau_{2})-L_{y}(\tau_{1})}{L_{y}(\tau_{1})},$$

$$\gamma_{yx}=\frac{L_{yx}(\tau_{2})-L_{yx}(\tau_{1})}{L_{y}(\tau_{1})}.$$ (Eq 10)

The ratio of the rotation angle to the length of the hindgut, represented by *r*_*θ*_, was given by:

$$r_{\theta }=2\pi \left(\frac{L_{yx}(\tau_{2})}{L_{x}(\tau_{2})}-\frac{L_{yx}(\tau_{1})}{L_{x}(\tau_{1})}\right).$$ (Eq 11)

The intercalation rate was calculated using a column of cells located in the middle (part of blue-colored cells, 23–28 cells per tube, Fig 4G). This rate was determined by dividing the number of cell boundaries diminished by cell intercalation by the total number of observed cell boundaries.

### Other mechanical factors possibly affecting hindgut extension and torsion

To concentrate on the effects of polarized contractions of cell boundaries, including anterior–posterior/distal–proximal anisotropy and chirality, our model made several simplifying assumptions. 1) The hindgut morphology was simplified to a cylinder. 2) Forces maintaining and deforming the hindgut were simplified to those from the apical junctions between cells, excluding external forces such as surrounding tissue and internal pressure. 3) Tissue viscosity was simplified to isotropic. While these simplifications help predict the effects of polarized contractions of cell boundaries, they may also affect hindgut extension and torsion. For instance, internal pressure, which counteracts epithelial extension, can delays the extension speed. Additionally, if tissue viscosity was anisotropic, it may either accelerate or decelerate the rate of extension and torsion.

## Supporting information

S1 Video Wild-type embryonic hindgut epithelium visualized using myr-GFP (membrane in green) and RedStinger (nuclei in magenta). (AVI)

S2 Video *E-cad* mutant embryonic hindgut epithelium visualized using myr-GFP (membrane in green) and RedStinger (nuclei in magenta). (AVI)

S3 Video Nuclear tracking in *E-cad* mutant hindgut epithelium using RedStinger. (AVI)

S4 Video Nuclear tracking in wild-type hindgut epithelium using RedStinger. (AVI)

S5 Video Wild-type embryonic hindgut epithelium overexpressing *Myo1D* visualized using myr-GFP (membrane in green) and RedStinger (nuclei in magenta). (AVI)

S6 Video Nuclear tracking in *Myo1D* overexpressing hindgut epithelium using RedStinger. (AVI)

S7 Video *Par-3 E-cad* mutant embryonic hindgut epithelium visualized using myr-GFP (membrane in green) and RedStinger (nuclei in magenta). (AVI)

S8 Video Nuclear tracking in *Par-3* mutant hindgut epithelium using RedStinger. (AVI)

S9 Video *ex vivo* cultured hindgut epithelium treated with DMSO visualized using myr-GFP (membrane in green) and RedStinger (nuclei in magenta). (AVI)

S10 Video *ex vivo* cultured hindgut epithelium treated with Y-27632 visualized using myr-GFP (membrane in green) and RedStinger (nuclei in magenta). (AVI)

S11 Video Nuclear tracking in *ex vivo* cultured hindgut epithelium treated with DMSO using RedStinger. (AVI)

S12 Video Nuclear tracking in *ex vivo* cultured hindgut treated with Y-27632 epithelium using RedStinger. (AVI)

S1 Fig Measurement methods of rotational movement index and elongation rate. *x–y* (A, B), *y–z* (A’, B’), and *z–x* (A”, B”) views of the hindgut at 0 min (A–A”) and 60 min (B–B”) after the beginning of the Video at late stage 12. Pivot lines were drawn from the middle of the lumen of the hindgut bottom (vertical white lines). The distance between the middle of the lumen at the peak of the elbow-shaped bend and the pivot line was measured (A horizontal white two-way arrow), and the difference in the distances between 0 and 60 min was defined as the rotational movement index. The length of the hook-like shape in the hindgut was also measured (a vertical white two-way arrow). The elongation rate was defined as the ratio of the length at 0 min to that at 60 min.
(TIF)

S2 Fig The *Par-3* mutant did not impair the LR asymmetric rotation of the hindgut. (A–C) The hindgut of *bowl* (A), *lines* (B), and *drumstick* (C) homozygotes visualized by UAS-*myrGFP* driven by *byn*-gal4. (D) Frequency of LR phenotypes in the hindgut of the *Par-3* homozygote. LR phenotypes are represented by colors shown on the left. Numbers on the top indicate the numbers of examined embryos. (E) *Par-3* homozygotes that showed a delay in germ band retraction exhibited largely normal hindgut rotation. In A, B, C, and E, the anterior is on top.
(TIF)

S3 Fig Changes in the DV depth of the hindgut during rotation and/or elongation. (A-C) Boxplots showing the DV depth of the hindgut in wild type (WT, N = 9) (A), *E-cad* mutant (N = 10) (B), and *Par-3* mutant (N = 5) (C) at 0 min (left) and 60 min (right) from the onset of rotation. (D) The DV depth ratios at 60 to 0 min in wild-type and *Par-3* mutant hindgut.
(TIF)

S4 Fig Simulations of gut deformation. Initial conditions (A-C) and final shapes (D-F) of the model tube in 2D resulting from simulations with (A,D) cell sliding only, (B,E) cell intercalation only, and (C,F) both simultaneously.
(TIF)

S5 Fig MyoII was required for the LR asymmetric rotation of the hindgut. (A) Still shots from a time-lapse Video of hindgut rotation in the *zip* mutant visualized as described in Fig 1A. The time elapsed from the start of the Video is shown on the lower right. (B) Frequency of LR phenotypes in the wild type (WT), *zip* heterozygotes overexpressing dominant-negative *zip* in the hindgut epithelium (driven by *byn*-gal4) (*zip*/+; *DN-zip*), and *sqh* homozygotes. LR phenotypes are represented by colors shown at the bottom. Numbers on the top indicate the numbers of examined embryos. (C) The hindgut visualized by UAS-*myrGFP* driven by *byn*-gal4 in the wild type (WT) and *zip*/+; *DN-zip* at stage 13. (D, E) Localization of Sqh-GFP in the wild-type hindgut epithelium driven by *byn*-gal4 at late stage 12 (D) and stage 13 (E). Brackets indicate the apical region of the hindgut epithelium. In A, C, E, and F, the anterior is on top.
(TIF)
