# Supplementary figures and images for: Distinct cellular and junctional dynamics independently regulate the rotation and elongation of the embryonic gut in Drosophila
Source: PLoS Genet. 2024 Oct 7;20(10):e1011422. doi: 10.1371/journal.pgen.1011422 (PMC11486408; doi:10.1371/journal.pgen.1011422)

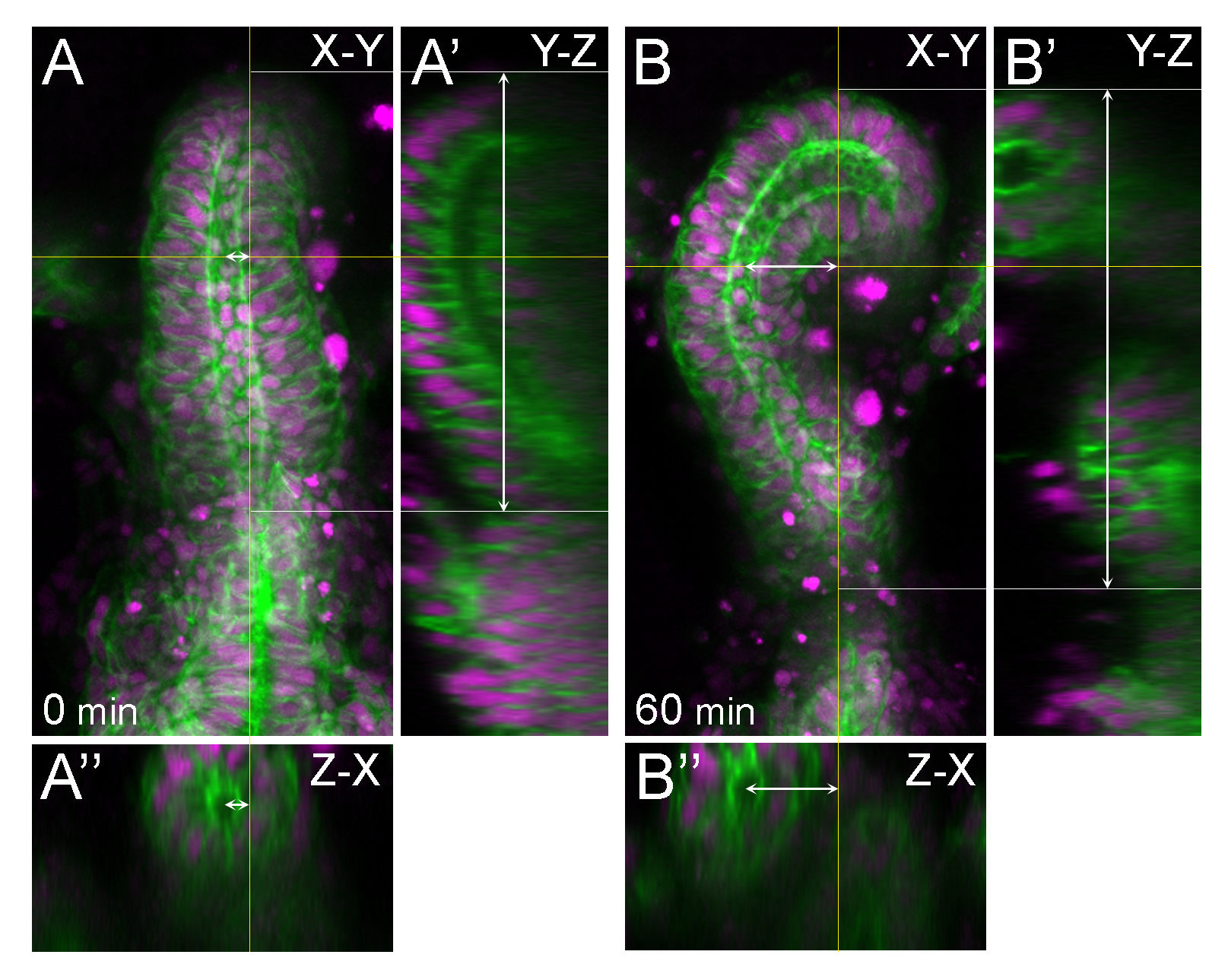

Supplement: S1 Fig — x–y (A, B), y–z (A’, B’), and z–x (A”, B”) views of the hindgut at 0 min (A–A”) and 60 min (B–B”) after the beginning of the Video at late stage 12. Pivot lines were drawn from the middle of the lumen of the hindgut bottom (vertical white lines). The distance between the middle of the lumen at the peak of the elbow-shaped bend and the pivot line was measured (A horizontal white two-way arrow), and the difference in the distances between 0 and 60 min was defined as the rotational movement index. The length of the hook-like shape in the hindgut was also measured (a vertical white two-way arrow). The elongation rate was defined as the ratio of the length at 0 min to that at 60 min. (TIF) [file pgen.1011422.s013.tif]

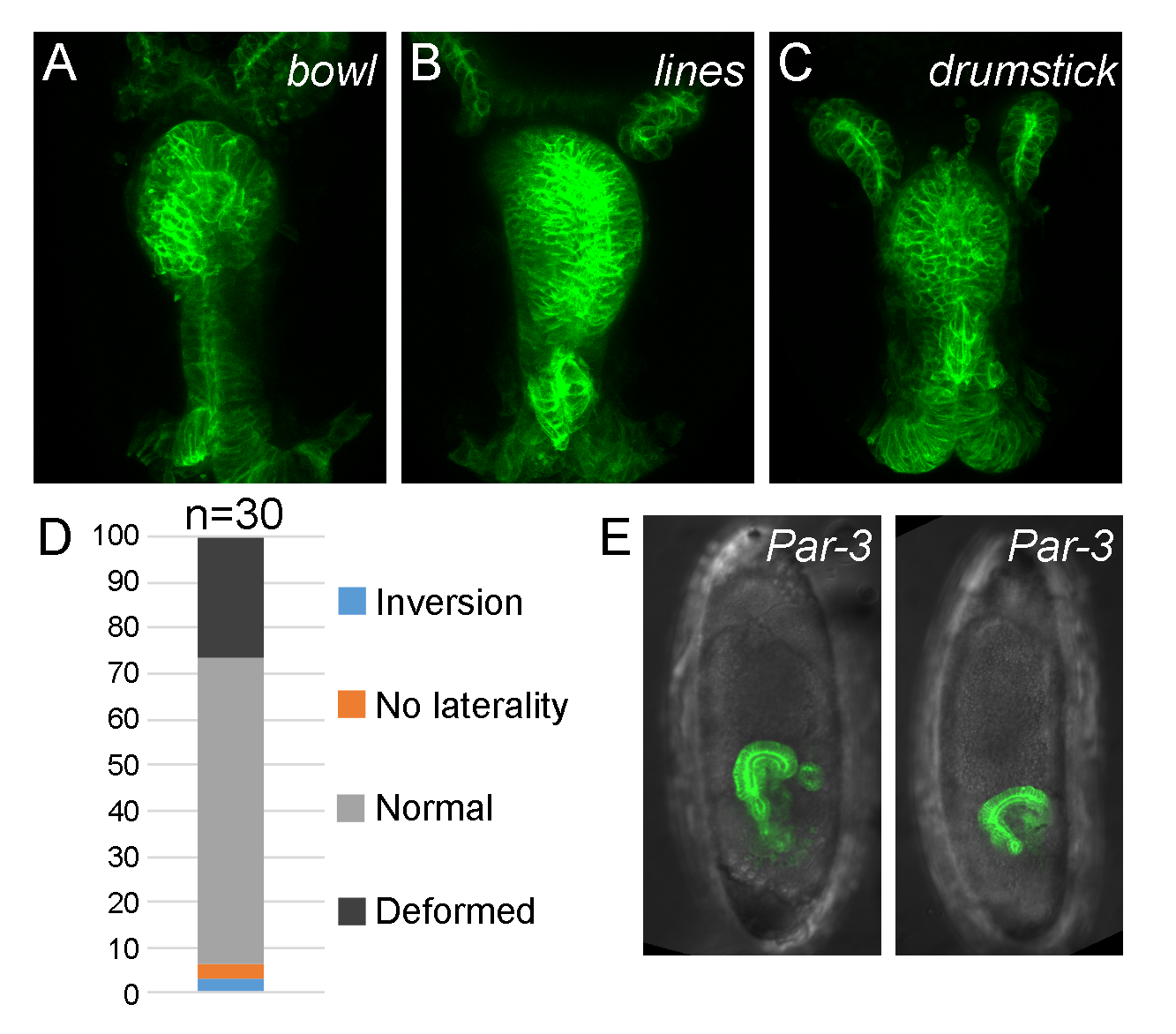

Supplement: S2 Fig — (A–C) The hindgut of bowl (A), lines (B), and drumstick (C) homozygotes visualized by UAS-myrGFP driven by byn-gal4. (D) Frequency of LR phenotypes in the hindgut of the Par-3 homozygote. LR phenotypes are represented by colors shown on the left. Numbers on the top indicate the numbers of examined embryos. (E) Par-3 homozygotes that showed a delay in germ band retraction exhibited largely normal hindgut rotation. In A, B, C, and E, the anterior is on top. (TIF) [file pgen.1011422.s014.tif]

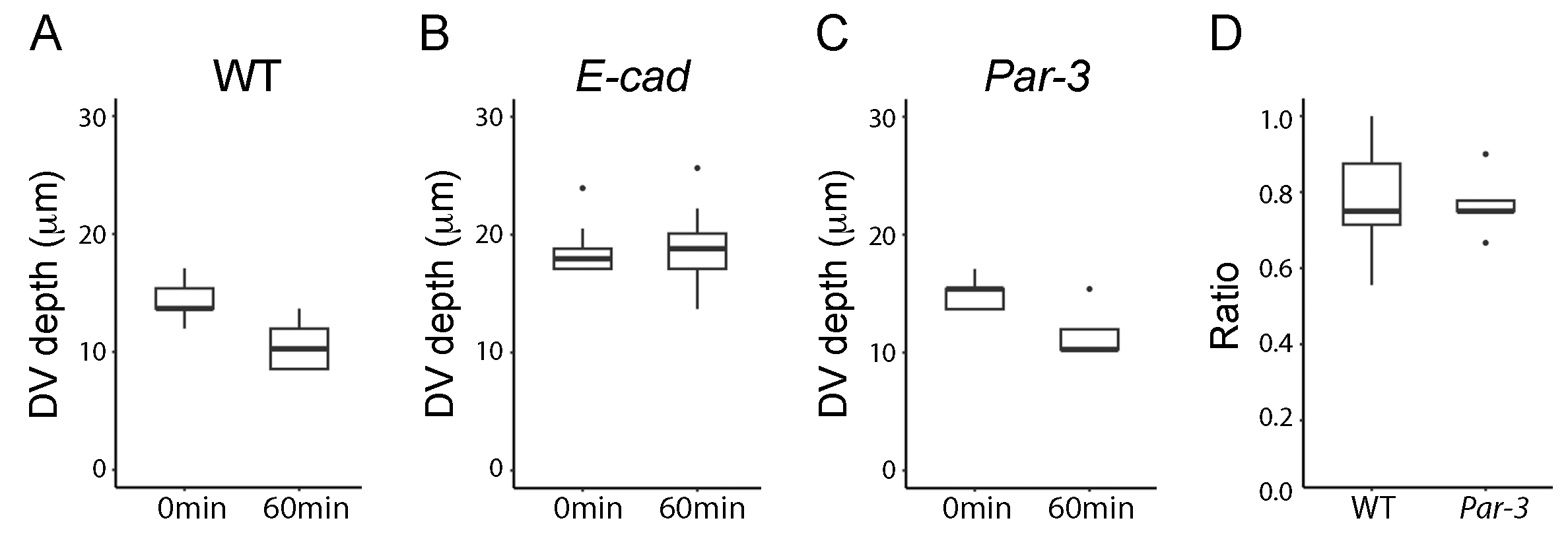

Supplement: S3 Fig — (A-C) Boxplots showing the DV depth of the hindgut in wild type (WT, N = 9) (A), E-cad mutant (N = 10) (B), and Par-3 mutant (N = 5) (C) at 0 min (left) and 60 min (right) from the onset of rotation. (D) The DV depth ratios at 60 to 0 min in wild-type and Par-3 mutant hindgut. (TIF) [file pgen.1011422.s015.tif]

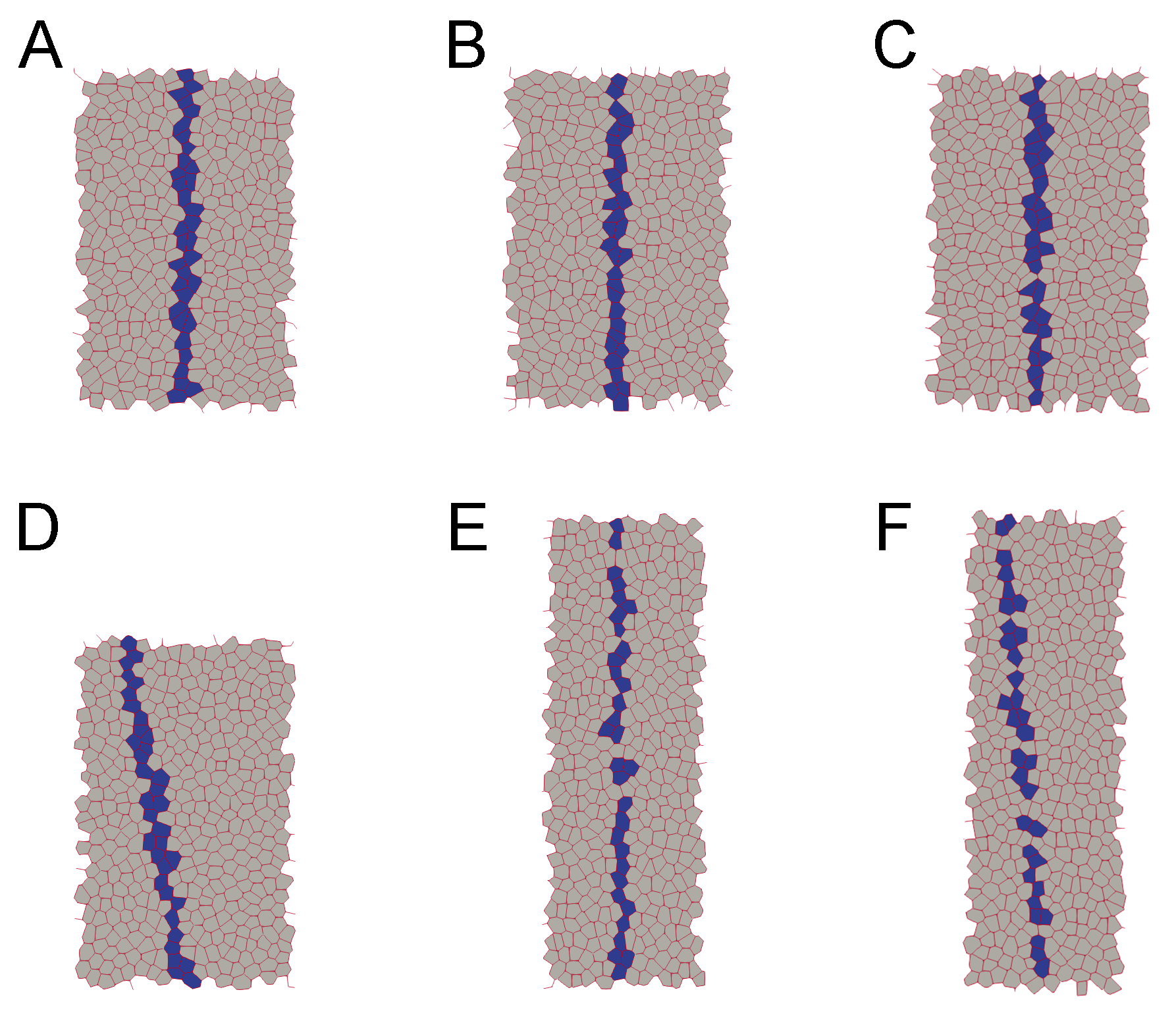

Supplement: S4 Fig — Initial conditions (A-C) and final shapes (D-F) of the model tube in 2D resulting from simulations with (A,D) cell sliding only, (B,E) cell intercalation only, and (C,F) both simultaneously. (TIF) [file pgen.1011422.s016.tif]

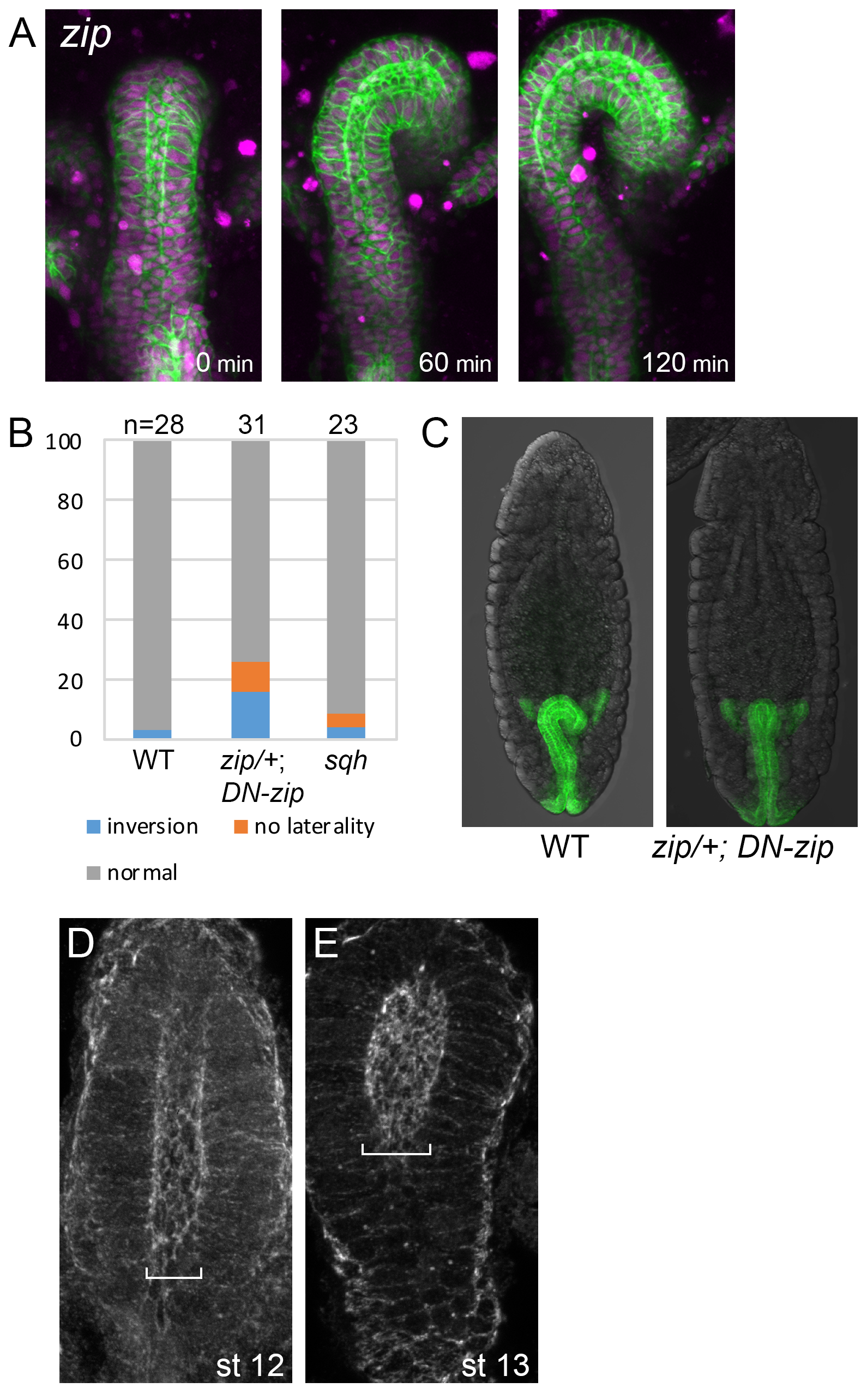

Supplement: S5 Fig — (A) Still shots from a time-lapse Video of hindgut rotation in the zip mutant visualized as described in Fig 1A. The time elapsed from the start of the Video is shown on the lower right. (B) Frequency of LR phenotypes in the wild type (WT), zip heterozygotes overexpressing dominant-negative zip in the hindgut epithelium (driven by byn-gal4) (zip/+; DN-zip), and sqh homozygotes. LR phenotypes are represented by colors shown at the bottom. Numbers on the top indicate the numbers of examined embryos. (C) The hindgut visualized by UAS-myrGFP driven by byn-gal4 in the wild type (WT) and zip/+; DN-zip at stage 13. (D, E) Localization of Sqh-GFP in the wild-type hindgut epithelium driven by byn-gal4 at late stage 12 (D) and stage 13 (E). Brackets indicate the apical region of the hindgut epithelium. In A, C, E, and F, the anterior is on top. (TIF) [file pgen.1011422.s017.tif]
