# Supplementary material for: Distinct cellular and junctional dynamics independently regulate the rotation and elongation of the embryonic gut in Drosophila
Source: PLoS Genet. 2024 Oct 7;20(10):e1011422. doi: 10.1371/journal.pgen.1011422 (PMC11486408; doi:10.1371/journal.pgen.1011422)
Supplement: Attachment — Submitted filename: 240718_response to revierwes.docx [file pgen.1011422.s018.docx]

Point-by-point response to

Reviewer #1: Major Comment

This manuscript follows up on the authors' previous works on the chiral rotation of the epithelial tube, the Drosophila hindgut. They previously showed that the rotation involves two steps. First, the epithelial cells in the gut tube, which itself is straight, take a chiral cell shape. In the second elongation step, the tube rotates while the cell shape asymmetry is resolved. In this work, the authors focused on the cell behavior during the tube elongation step. They attempted to dissect elements of cell shape change and their contribution to the tube rotation. Using genetic and pharmacological perturbation experiments, quantitative measurement, and mathematical modeling, the authors suggested two cell behavior elements, “cell sliding” and “convergent extension,” contribute differently to the tube rotation. I should raise three issues and ask the authors to resolve them.

1. To argue the independence of the two elements, “cell sliding” and “convergent extension,” each value should represent distinct, independent cell properties. Cell sliding was defined as shifting the left-right position of a pair of adjacent cell nuclei. Formally, the shift can result from a simple cell position change with a conserved cell interface. Alternatively, the shift may involve the sliding of cell boundaries with changes in cell interface relationship (intercalation). As the authors demonstrated, cell intercalation is the major driver of convergent extension. Since cell intercalation could also be involved in cell sliding, the two values cannot be considered independent. For the argument to stand, the authors should provide evidence that the two values are separate measures of cell behavior and present them before they start arguing about possible relationships of the values.

Response: Thank you for the insightful comments. As highlighted by Reviewer #1, it has been noted that cell sliding potentially causes junctional remodeling and since both cell sliding and intercalation occur simultaneously, formally separating these processes poses a challenge. However, we propose that cell sliding without junctional remodeling contributes to the left-right asymmetric rotation of the hindgut for the following reasons, as demonstrated in our previous study (Inaki et al., 2018): first, most cell junctions remain intact during cell sliding. Second, in both the wild-type and inverted rotation scenarios, there is no observed left-right bias in cell intercalation. Third, the model without junctional remodeling successfully recapitulated cell sliding and hindgut rotation. Therefore, the shift in cell nuclei is not caused by the sliding of cell boundaries but rather by chiral changes in cell shape. In this study, we further demonstrated that cell sliding (x displacement) can be genetically separated from cell intercalation. To clarify these points, we have added the following sentences to the Results section of the revised manuscript:

P13 L201

Junctional remodeling has been considered unrelated to cell sliding or hindgut rotation because most cell boundaries were maintained during cell sliding, and the cell boundaries diminished by junctional remodeling did not exhibit LR bias^22^. Additionally, a simulation without junctional remodeling successfully recapitulated hindgut rotation^22^.

2. The authors measured the tube length from 3D stacks of live imaging data. If I understand correctly, the length value was taken from the straight line between the anterior-most edge of the tube and the author-defined posterior starting point (Fig. S1). The ideal measure for the tube length should be the length of the tracked tube lumen, which is curved in 3D space. I need clarification on the rationale of using straight lines to represent the length of curved and rotated tubes.

Response: Thank you for the constructive comments. As highlighted by Reviewer #1, ideally, the length of the gut tube should be measured in 3D due to its curvature. To address this point, we attempted to measure the length of the tracked lumen in the gut tube. However, we encountered technical challenges in making such measurements. In our analyses, we assumed that the straight line between the anterior-most edge of the tube and the author-defined posterior starting point should increase if the gut tube elongates through convergent extension. However, as noted by Reviewer #1, there is a potential issue if the gut tube leans ventrally, as elongation using our procedure may not be detectable. To mitigate this concern, we measured the depth of the gut tube along the dorsal-ventral axis in the revised manuscript. In *Par-3* mutant hindgut, we observed a reduction in the elongation of the gut tube, as evaluated using our procedure. In the revised manuscript, we measured the depth of the gut tube along the dorsal-ventral axis to exclude the possibility that the reduction in elongation was due to leaning ventrally. We found that the depth in *Par-3* mutant is equivalent to that of the wild type. Furthermore, the ratios of the depth 0 min to 60 min rotation were similar between *Par-3* mutant and the wild type, further excluding the ventral extension of the gut tube in *Par-3* mutant. Therefore, we conclude that the reduction in gut tube elongation in *Par-3* mutant reflects the decrease in elongation along the anterior-posterior axis. Thus, our procedure provides an approximate assessment of gut tube elongation. To present these new experiments, we have included the results in Figure S3A-D of the revised manuscript. Additionally, we have added the following sentences to the Results, Materials and Methods, and Figure legends to describe these results:

P16 L263

However, the observed reduction in hindgut elongation detected in our assay could be explained if the hindgut leaned ventrally. To exclude this possibility, we measured the depth of the hindgut along the dorsal-ventral axis (DV depth) at 0 and 60 min (Figure S3A-D). Our results demonstrated that DV depth was similar between wild type and *Par-3* mutant hindgut, and the ratios at 0-60 min were equivalent for both genotypes (Figure S3A, C, and D). Conversely, the DV depth of *E-cad* mutant differed from that of the wild type due to suppressed hindgut rotation (Figure S3B). These findings indicate that wild-type Par-3 regulates hindgut elongation through cell intercalation without affecting cell sliding and hindgut rotation.

P31 L533

The DV depth was measured as the distance between the most dorsal part of the gut lumen and the most anterior peak of the hook-like structure along the z (dorsal-ventral-axis) direction at 0 and 60 min (Figure S1 and S3A-D).

P 54 L988

Figure S3

Changes in the DV depth of the hindgut during rotation and/or elongation. (A-C) Boxplots showing the DV depth of the hindgut in wild type (WT, N=9) (A), *E-cad* mutant (N=10) (B), and *Par-3* mutant (N=5) (C) at 0 min (left) and 60 min (right) from the onset of rotation. (D) The DV depth ratios at 60 to 0 min in wild-type and *Par-3* mutant hindgut.

3. The authors compared the tube elongation behavior in control, E-cad mutant and MyoID overexpression. Those genetic perturbations can also influence the first step of cell chirality before cell elongation. If this is the case, the elongation movement started from different starting conditions. How can the authors argue that E-cad and MyoID overexpression differently affect cell behavior at the time of elongation, not before?

Response: Thank you very much for all the comments. We have noticed some confusion here. In this manuscript, we reported that *E-cad* mutant and *MyoID* overexpression did “not” affect elongation. Therefore, *E-cad* mutant and *MyoID* overexpression did “not” affect cell behavior before elongation.

Other Comments

Abstract. “simultaneous” was used 6 times.

Response: Thank you for your comment. We have revised the previous abstract and minimized the use of “simultaneous” to three instances as follows:

P2 L20

In this study, we revealed how distinct shape changes are controlled during internal organ morphogenesis. The *Drosophila* embryonic hindgut undergoes left-right asymmetric rotation and anteroposterior elongation in a tissue-autonomous manner driven by cell sliding and convergent extension, respectively, in the hindgut epithelia.

Line 24. “we demonstrated that independently regulated cellular dynamics can simultaneously cause shape changes in a mechanical system.”　The sentence does not seem to provide meaningful information. However, how they are regulated remains unclear.

Response: Thank you for your comments. We aim to assert that two tissue deformations caused by different cellular behaviors can be recapitulated by a single equation of motion using anisotropic edge contraction. To clarify this point, we have revised the following sentences in the Abstract:

P2 L24

Through genetic analysis and live imaging, we demonstrated that cell sliding and convergent extension are independently regulated by Myosin1D and E-cadherin, and Par-3, respectively, whereas both require MyosinII activity. Using a mathematical model, we demonstrated that independently regulated cellular dynamics can simultaneously cause shape changes in a single mechanical system using anisotropic edge contraction.

Line 37 “Organ shape is closely related to its functions; therefore, it needs to be precisely controlled during development1.” Difficult sentence. Maybe “Organ shapes are optimized to execute their function.”

Response: Thank you for the comment. We have amended the sentence as suggested.

P4 L38

Organ shapes are optimized to execute their functions.

Line 38 What is indicated by “Epithelial morphology” here?

Response: Thank you for your comment. We meant to convey that epithelium is responsible for the shaping. Therefore, we have changed the words “Epithelial morphology” to “Epithelium”.

Abstract and Introduction. The words “simultaneous” and “coordinate” are used to describe the relationship of more than two events (or parameters). What was compared was inconsistent. In the introduction, cellular vs molecular mechanisms were compared. In the abstract, anteroposterior elongation and left-right shifting are compared.

Response: Thank you for your comments. We would like to compare the anteroposterior elongation and left-right shifting in both Abstract and Introduction. We have recognized that the misuse of the words “machineries” and “mechanisms” has caused confusion. We have corrected the word usage in the following sentences in the Introduction section:

P4 L42

Over the past decade, the cellular and molecular bases underlying each mechanism in epithelial morphogenesis have become well understood, although they have been studied separately in simplified systems. However, it is hypothesized that complex epithelial morphology results from the simultaneous and coordinated functioning of these morphogenetic mechanisms. This implies that the mechanical processes governing tissue morphogenesis can be delineated into distinct setups that collaboratively control the dynamic behaviors of a single tissue. However, the potential interactions or independence among these mechanisms remain unclear.

P6 L87

Convergent extension and chiral cell sliding contribute simultaneously to the morphogenesis of the hindgut epithelium, making the hindgut an excellent system for studying the potential independence and relevance of these two morphogenetic mechanisms.

P7 L94

Furthermore, mathematical models of hindgut morphogenesis were constructed to evaluate the individual and integrated contributions of these mechanisms. Our analyses revealed that while convergent extension and chiral cell sliding are separable events, they share common machinery that is utilized differently in the polarized contraction of cell boundaries: anterior-posterior/distal–proximal anisotropy and chirality. We found that these mechanisms can independently and concurrently induce convergent extension and chiral cell sliding, respectively. Therefore, our results demonstrate that the concurrent actions of these distinct morphogenetic mechanisms are crucial for the complex transformation of organ shapes.

Line 202 “This transition encourages these epithelial cells to intercalate with each other,“ encourage may be replaced by drive, promote, etc.

Response: Thank you for your comment. We have amended the sentence as suggested.

P13 L200

This transition drives these epithelial cells to intercalate with each other, resulting in the elongation of the hindgut epithelial tube.

Reviewer #2: The authors presented an intriguing study on the simultaneous shape changes in Drosophila embryonic hindgut morphogenesis, focusing on cell sliding and convergent extension. The integration of genetic analysis, live imaging, and mathematical modeling offers valuable insights into complex organ formation. However, there are several areas where clarification and additional information are required to support the findings and conclusions more robustly.

1. Clarification on Contractions and Equations (P17 L275):

Please clarify the relationship between contractions with chirality and anterior-posterior/distal-proximal anisotropy and Equations 4 and 5. How are these phenomena represented within the mathematical model?

Response: Thank you for your comments. Both chirality and anterior-posterior/distal-proximal anisotropy are introduced via the third term of Equation (4), but they are introduced at different times. In this term, the line tension coefficient includes a term that depends on the angle of cell boundary, as shown in Equation (5). Specifically, chirality is represented by biased cell shapes under the initial condition, which was induced by setting $\theta_{\mathrm{ref}}$ to 45 degrees in the precalculation. Conversely, anterior/posterior/distal-proximal anisotropy was represented by biased edge contraction induced by setting $\theta_{\mathrm{ref}}$ to 0 during the simulation. To clarify these points, we have added the following sentences in the Material and Methods section:

P35 L607

This term represents both polarized contractions of cell boundaries, including anterior–posterior/distal–proximal anisotropy and chirality. The detailed implementation of these polarized contractions is described in the following simulation procedure.

P37 L634

During the precalculation, the angle of the polarized contraction of cell boundary was set to $\theta_{\mathrm{ref}}=\pi/4$ to represent the chirality causing chiral cell sliding. Additionally, during torsion, the angle was set to $\theta_{\mathrm{ref}}=0$ to form the anterior–posterior/distal–proximal anisotropy of cell shapes, which drives convergent extension.

2. Reference to Equations for Axial and Radial Expansion (P17 L282):

Is the consideration 'axial and radial expansion and contraction of the hindgut' referring to Equation 2?

Response: Thank you for your comment. The axial and radial deformation of the hindgut refers to the first and second equations of (1), respectively. To clarify this point, we have amended the sentence in the Results section as follows:

P18 L289

In this study, we enhanced this model by incorporating the axial and radial expansion and contraction of the hindgut, which facilitates hindgut elongation (refer to equations (1) in Materials and Methods).

3. Question on Two Oriented Angles (P17 L287):

In the section discussing 'edge tension biased at two oriented angles' (Fig4E), could you specify what is meant by 'two oriented angles'? The figure appears to depict only one angle. Additionally, could you explain the concept of 'left-right-biased contraction' mentioned in the caption, and how it relates to the contractions with chirality and anterior-posterior/distal-proximal anisotropy introduced earlier?

Response: Thank you for all the comments. We have realized that Figure 4E only illustrates angle-dependent edge contraction. When referring to ‘Two oriented angles’, we meant 45 degrees (rightward tilted edge) for chirality and 0 degrees (horizontal edge) for anterior-posterior/distal-proximal anisotropy. The description of ‘Anterior-posterior/distal-proximal’ anisotropy in the same sentence caused confusion. In the caption, 'Left-right-biased contraction' referred to the edge contraction of rightward tilted edge for chirality formation in the top left of Figure 4F. However, this explanation was not appropriate for Figure 4E. To clarify these points and avoid confusion, we have amended the sentences in the Results and Figure legends as follows:

P18 L294

Anisotropic contraction of the cell boundaries was introduced as edge tension biased at oriented angles (Figure 4E).

P50 L923

(E) Biased contraction, dependent on edge angle was introduced (indicated by two arrows). (F) To recapitulate cell sliding, initial cell chirality was formed by rightward-tilted-edge biased contraction and its subsequent dissolution were introduced (upper).

4. Clarification on Cell Chirality Introduction (P18 L288):

The text states 'Cell chirality was introduced as cell shape strain under the initial condition.' Is this referring to the cell shapes shown in Figure 4G?

Response: Yes, it is. The illustration of cell chirality is depicted in the top left of Figure 4F, while the dissolution of cell chirality, which causes cell sliding is shown in top right of Figure 4F. To clarify these points, we have revised the sentences in the Results section as follows:

P18 L296

Cell chirality was represented by initial cell shape strain (Figure 4F top left, 4G), induced by biased edge tension maximal at 45 degrees from the *x*-axis. As the simulation progressed, this tension was dissolved (Figure 4F top right) and a horizontal biased edge tension, maximal at 0 degrees was added (Figure 4F bottom).

5. Chirality in E-cad-mutant Cells and Simulations (P18):

The authors mentioned that cell chirality was found to be abolished in E-cad-mutant cells (p8 L120). In the simulations under E-cad-null conditions (Fig4I), is chirality assumed initially similar to other simulations? If so, why doesn't Fig4I seem to show the expected disappearance of chirality followed by cell sliding?

Response: Thank you for the comments. We have not previously presented the image of the initial condition for *E-cad* null in Figure 4. Specifically, the initial condition of the *E-cad* null mutant does not exhibit chirality or demonstrate a disappearance of chirality followed by cell sliding. To clarify this point, we have added initial and final images of all the conditions in Supplemental Figure 4.

6. Frequency of Cell Intercalation in Simulations vs. Experimental Observations:

It's noted that cell intercalation occurs at approximately a 10% frequency in experiments. Do the simulations replicate this frequency, particularly in explaining the elongation rate? It would be important to verify whether the model's assumptions about cell intercalation frequency align with experimental observations to support the model's validity.

Response: Thank you for the valuable comments. We realized that our models in the previous manuscript could not successfully recapitulate the intercalation rate observed *in vivo* and that the shapes of the cells after tissue deformation appeared more angular than those observed. To address this, we modified and added parameters based on published literature (Farhadifar et al, 2007). Specifically, Peq was set to 0 and an additional coefficient, $\Lambda_{c}$ for edge contraction, was introduced (Farhadifar et al, 2007). Using this revised model, we successfully recapitulated the hindgut rotation and elongation as well as the frequency of cell intercalation. In the wild-type hindgut, intercalation occurs in approximately 10% of cell boundaries every 30 minutes. Given that hindgut completion takes approximately 2 hours, and assuming a constant intercalation rate, intercalation events will occur in approximately 40% of cell boundaries over the entire elongation period. The average intercalation rate from five simulations of the wild-type hindgut was determined to be 35.4%. For the *E-cad* mutant simulations, intercalation rates were slightly lower but largely comparable to the *in vivo* results. Furthermore, we used the revised model to recapitulate the effect of drug treatment inhibiting MyosinII activity by eliminating edge tension during the tissue deformation period. However, the complete loss of edge tension caused isotropic expansion of the tissue, which did not parallel the *in vivo* data. Consequently, we hypothesized that the complete elimination of edge tension could not be achieved by the drug treatment, and thus used conditions of reduced edge tension. The simulations with reduced tension showed decreases in rotation and elongation, qualitatively recapitulating the experiments. These results have been incorporated into Figure 4M, while Figure 4G–L and Figure 5I–K have been replaced. Corresponding changes and additions have been made to the Results, Material and Methods, Table 3 and Figure legends as follows:

P19 L318

In the wild-type hindgut, cell intercalation occurred at 9.3% of the cell boundaries every 30 min (Table 1). Consistent with these observations, the wild-type model exhibited an average cell intercalation rate of 35.4% over a rotation and elongation period of approximately 2 h *in vivo* (Figure 4M).

P23 L381

We next validated these results in our *in silico* model. We concurrently diminished the dissolution of cell chirality and contractions of cell boundaries with anterior–posterior/distal–proximal anisotropy by reducing edge tensions in the model, although chirality was introduced before edge tension reduction (Figure 5I). We found that this model gut showed decreases in rotation and elongation (Figures 5I–K), which is similar to the findings in the hindgut in which MyoII activity was inhibited *ex vivo* (Figures 5C, 5E, 5G, and 5H). Thus, our *in silico* model qualitatively recapitulated the situations.

P36 L612

$$\Lambda_{j}={\Lambda_{c}+\Lambda}_{a}\cos2\left( \theta_{j}-\theta_{\mathrm{ref}} \right)+\Lambda_{r}w_{j},$$

P38 L642

For the simulation using reduced tension, $\Gamma$ and $\Lambda_{a}$ were reduced by 0.01 holds and $\Lambda_{r}$ and $\Lambda_{r}$were reduced by 0.1 holds in torsion.

P38 L653

The intercalation rate was calculated using a column of cells located in the middle (part of blue-colored cells, 23-28 cells per tube, Figure 4G). This rate was determined by dividing the number of cell boundaries diminished by cell intercalation by the total number of observed cell boundaries.

P45 L853 in Table 3

| $P_{\mathrm{eq}}$ |  | 0 | 0 | ^38^ |
| --- | --- | --- | --- | --- |
| $\Lambda_{c}$ | $K\left( A_{\mathrm{eq}} \right)^{3/2}$ | 0.12 | 0.12 | ^38^ |

P51 L934

(K, L, M) Quantification of rotation angle, elongation, and intercalation rates was performed *in silico* from 5 independent simulations for each condition. (K) Boxplots of rotation angle (degree). (L) Boxplots of elongation rate. (M) Boxplots of intercalation rate (%). Black dots are outliers.

P52 L957

Results of simulation in which edge tension was reduced after the initial introduction of chirality.

P52 L960

(J, K) Boxplots of rotation angle (degree) (J) and extension rate (K) in models implementing both cell sliding and convergent extension with normal (control) or reduced (low tension) edge tensions.

7. Model's Representation of Cell Boundary Contractions (P18 L292):

It is unclear which parts of the equations correspond to 'cell boundary contractions with chirality or anterior–posterior/distal–proximal anisotropy' as they are introduced separately in the model. Could you provide further explanation in Materials and Methods?

Response: Thank you for the comments. To provide clarification, we have explained and added sentences similar to our response to comment #1.

8. Directionality and Figure References (P18 L295, L297, L299):

Regarding 'in the counterclockwise direction' (Figure 4G), should this refer to Fig4H? Given the right-hand coordinate system, would the direction be clockwise if the z-axis orientation is considered?

It seems there might be confusion in figure references: Fig4H seems to be referred to as Fig4I, and Fig4I as Fig4J. Could you clarify these references?

Response: Thank you for all the comments. To define the directions of hindgut rotation, we have consistently used the view from the posterior end *in vivo* (the view from the bottom of the tube in the model), where the direction of hindgut rotation is counterclockwise. To clarify this point, we have revised the following sentences in the Results section and corrected the figure references:

P18 L301

The introduction and subsequent dissolution of chiral tension caused the model tube to rotate approximately 90 degrees counterclockwise, as viewed from the bottom of the tube (Figure 4H). Additionally, applying edge tension with anterior–posterior/distal–proximal anisotropy resulted in axial elongation of the model tube (Figure 4I, S4). Notably, integrating both tensions led to simultaneous tube rotation and elongation, effectively recapitulating hindgut morphogenesis *in vivo* (Figure 4J, S4).

9. Request for Detailed Model Explanation and Equation Clarification:

The explanation of the model and its underlying equations appears overly simplified, making it difficult to grasp the essential details. A more detailed explanation is requested.

9-1. Specifically, Equation 2 seems to lack boundary condition terms that would ensure equilibrium states conform to physical laws, such as Laplace's law.

Response: Thank you for the comment. In the previous manuscript, we did not provide sufficient explanation of the boundary conditions. We have now added the following explanation of the boundary conditions in the Material and Methods section:

P32 L554

The multicellular dynamics within the cylinder were calculated by expanding them to those in a rectangular sheet of width $L_{x}$ and length $L_{y}$ in the *xy*-coordinates, where the radial and longitudinal axes of the cylinder correspond to the *x-* and *y-*axes of the sheet, respectively. Periodic boundary conditions were applied at boundaries $x=L_{x}$ and $y\boldsymbol{=}L_{y}$. To represent the longitudinal extension of the cylinder, the perimeter $L_{x}$ and length $L_{y}$ of the cylinder can vary depending on the tissue stress. Additionally, to represent the torsion of the cylinder, the $x$-coordinate can be shifted at the boundary from $y=L_{y}$ to $y=0$ with length $L_{yx}$ according to the tissue stress.

9-2. For the circumferential direction, the equilibrium state should balance the external pressure difference P across the tube's wall and the circumferential tension, determining the radius R, (represented here as the circumferential length Lx). The equation might be represented as:

xi dLx/dt = -Lx sigma_xx + Lx^2 P/h where h is the cell height (along apicobasal axis).

Similarly, for the axial direction, considering the tube is connected to surrounding tissues, the boundary stress could be represented as sigma_n, leading to:

xi dLy/dt = -Ly(sigma_yy – sigma_n)

These considerations raise questions about the stability of cylindrical shapes under the authors' original equations, particularly if they do not reconcile with Laplace's law at equilibrium, suggesting that the cylindrical shape visualized in Fig4 might not be stable under the author’s proposed condition (P=0).

Response: Thank you for all the comments. In the previous manuscript, we did not adequately explain several assumptions that simplified the model of the hindgut, focusing instead on the effect of anisotropic boundary contractions. We only considered the apical surfaces of hindgut epithelial cells, where adherence junctions exist. We previously found that hindgut rotation occurs independently of other organs. Thus, we did not consider surrounding tissues. To clarify these points, we have revised and added the following sentences in the Materials and Methods section:

P32 L549

**Two-dimensional (2D) vertex model for hindgut extension and torsion**

To analyze the effects of polarized contractions of cell boundaries, such as anterior–posterior/distal–proximal anisotropy and chirality, on hindgut deformation, we performed numerical simulations of epithelial torsion by enhancing a 2D vertex model.

P33 L563

The rectangular sheet forming the cylinder is densely lined with numerous cells.

P33 L566

Cell dynamics were calculated by tracking the motion of vertices, where the location vector of the *i*th vertex is represented by $\boldsymbol{r}_{i}$. In addition, cell rearrangement was represented by reconnecting the edges when the length of the edges fell below a threshold value $\Delta l$, based on the motion of vertices.

P33 L572

To combine cylindrical deformation with multicellular dynamics, we followed a sequential calculation process: first, we calculated the geometry of cells $\boldsymbol{r}_{i}$, from which we obtained the stress tensor of the entire tissue, represented by $\sigma_{\alpha\beta}$. Using this stress tensor, we then calculated the geometry of the cylinder $L_{x}$ and $L_{y}$. The iterative calculations of these processes allowed us to calculate the combined dynamics of the cylinder and cells.

P34 L571

For simplicity, we ignored the effects of epithelial thickness.

P34 L584

**Motion equation of a deformable tube**

Hindgut torsion occurs independently of surrounding tissues (Inaki et al, 2018). Additionally, abnormalities in chirality and torsion can be rescued by expressing Myosin1D in the posterior intestinal epithelium (Taniguchi et al., 2011, Hozumi et al, 2006). These findings suggest that hindgut torsion is driven autonomously by actomyosin-dependent junctional contraction of the hindgut epithelium. Therefore, we focused on the effects of junctional contractions, disregarding external forces such as luminal pressure and other forces from the hindgut environment.

P39 L659

To concentrate on the effects of polarized contractions of cell boundaries, including anterior–posterior/distal–proximal anisotropy and chirality, our model made several simplifying assumptions. 1) The hindgut morphology was simplified to a cylinder. 2) Forces maintaining and deforming the hindgut were simplified to those from the apical junctions between cells, excluding external forces such as surrounding tissue and internal pressure.

9-3. The authors' original formulation for the circumferential direction seems to lack an explicit representation of these dynamics, potentially overlooking how the equilibrium tube radius R (Lx) is determined by the balance of forces.

It is believed that this could have a significant impact on convergent extension.

Therefore, additional discussion on this aspect would be highly beneficial.

Moreover, the application of isotropic linear elasticity, as possibly assumed in deriving Equation 3, to conditions leading to plastic deformations like convergent extension warrants further discussion.

Response: Thank you for the comments. To emphasize the effects of polarized contractions of cell boundaries, such as anterior-posterior/distal–proximal anisotropy and chirality, our model simplified the phenomenon under several assumptions. These include considering the hindgut as a simple cylinder and isotropic viscosity. To explain these points, we have added a section in the Material and Methods as follows:

P39 L658

# **Other mechanical factors possibly affecting hindgut extension and torsion**

To concentrate on the effects of polarized contractions of cell boundaries, including anterior–posterior/distal–proximal anisotropy and chirality, our model made several simplifying assumptions. 1) The hindgut morphology was simplified to a cylinder. 2) Forces maintaining and deforming the hindgut were simplified to those from the apical junctions between cells, excluding external forces such as surrounding tissue and internal pressure. 3) Tissue viscosity was simplified to isotropic. While these simplifications help predict the effects of polarized contractions of cell boundaries, they may also affect hindgut extension and torsion. For instance, internal pressure, which counteracts epithelial extension, can delays the extension speed. Additionally, if tissue viscosity was anisotropic, it may either accelerate or decelerate the rate of extension and torsion.

9-4. Also, the role and significance of zeta in Equation 4 are not described. Clarification on its meaning and the rationale for not assuming linear elasticity (zeta = 0) would provide valuable insights for readers.

Response: Thank you for the comments. Zeta, which represents the constraint strength, was introduced to prevent excessive shrinkage and expansion of the cell. To elaborate on the role and significance of zeta, we have added the following sentence in the Material and Methods:

P35 L600

The coefficient $\zeta\left( 1-A_{\mathrm{eq}}/A_{i} \right)^{2}$ in the first term regulates the individual cell size, preventing excessive shrinkage or expansion, where $\zeta$ represents the constraint strength.

Reviewer #3: Inaki, Matsuno and colleagues study the cell biology of the development of the gut in the Drosophila embryo, using wt and mutant strains. This model system is particularly interesting and relevant as it combines asymmetric rotation by cell sliding and AP elongation by convergent extension (CE). The authors impressively demonstrate that both processes are independently regulated by the actin-based molecular motor MyoID and the adhesion protein E-cad on the one hand (cell sliding) and the cell polarity regulator Par3.

The results are well presented, tightly controlled and of high quality. This is particularly

true for the movies. Although I tried hard I did not find absolutely necessary additional experiments to be performed.

Due to lack of competency, I could not judge the model.

1. What I, however, truly missed is a proper discussion. What is presented is in large parts a repetition of the results. How do these results relate to the published literature? What do they mean in an evolutionary context?

Response: Thank you for the comments. To describe the relationship with published literature, we have added the following sentences in the Discussion section:

P27 L458

Cell intercalation associated with LR asymmetric morphogenesis has been reported in the development of the mouse and Xenopus stomachs, and the zebrafish heart ^53,54^. In mouse and *Xenopus* stomachs, radial cell rearrangement expands the left side wall more than the right, causing the stomach tube to bend leftward as if rotating ^53^. In the zebrafish heart tube, cells on the left intercalate more actively than those on the right, thereby driving the clockwise rotation of the heart tube ^54^. In these cases, cell intercalation triggers LR asymmetric morphogenesis. However, in the *Drosophila* embryonic hindgut, cell intercalation does not affect LR asymmetry but instead causes tissue elongation. Therefore, this indicates that cell intercalation in LR asymmetric development is evolutionarily divergent. Additionally, tissue elongation in the anterior-posterior (cranial-caudal) direction may enhance LR asymmetric morphogenesis in confined body cavities. In chick embryos, gut tube elongation following LR asymmetry determination by the dorsal mesentery induces LR asymmetric looping of the organ ^1,55^. During heart tube development in chick embryos, tissue elongation enhances the looping structure ^1^. However, as reported in this study, in the *Drosophila* embryonic hindgut of the *Par-3* mutant in which tissue elongation was absent, the extent of LR asymmetry in this organ was comparable to that in the wild type, indicating that tissue elongation does not enhance LR asymmetry. Therefore, the independent regulation of LR asymmetric rotation and anterior-posterior elongation in the *Drosophila* hindgut suggests that divergent mechanisms exist in morphogenesis associated with LR asymmetry formation of internal organs among species.

2-1. -minor language issues (line 16 add ‚responsible’ before epithelium; add Myo1D to Keywords;

Response: Thank you for the comment. We have added ‘responsible’ before epithelium and Myo1D to the Keywords.

2-2. non-drosophilists may have problems to follow the name of mutants (i.e. shg for E-cad);

Response: We have substituted the gene names *shg* and *baz* with E-cad and *Par-3*, respectively, in the following sentences in the Results:

P8 L125

However, the *E-cad*-mutant hindgut of the hardly rotated under this condition (Figures 1B1–3, Video 2).

P9 L128

After 30 and 60 min, the relative positions of the nuclei did not change markedly in *E-cad*-mutant embryos (Figures 1E1–3, Video 3); however, the rows of nuclei slanted to the left in the wild type, as reported previously (Figures 1D1–3, Video 4).

P12 L183

In the *E-cad* mutant, the rotational movement index was almost negligible.

P13 L210

In the *E-cad* mutant, in which cell sliding was largely abolished, the cell intercalation was still observed at a frequency of 12.7% in the same interval, supporting the independent regulation of these two cellular dynamics (*shg^69B^* in Table 1, Figure 2F).

P15 L235

Compared with the wild type, we found that the LR bias of cell boundary rotation largely disappeared in the *E-cad* mutant (Figure 2K).

P16 L260

However, their elongation rate was significantly reduced in the *Par-3*/*baz* mutant (Figures 3C and 3F), which coincided with a severe decrease in the cell intercalation to a frequency of 2.0% compared with 9.3% in the wild type (Table 1).

2-3. line 152: occasionaly = almost 50%

Response: Thank you for the comments. The term ‘occasionally’ was used to indicate a frequency close to 50%. However, in the following sentence (L151) ‘often’ was deemed unsuitable and has been removed.

3. First paragraph of Intro: which ‘hypothesis’ is eluded to (line 46)?

Response: Thank you for the comment. To eliminate ambiguity, we have revised the sentences in the Introduction as follows:

P4 L45

However, it is hypothesized that complex epithelial morphology results from the simultaneous and coordinated functioning of these morphogenetic mechanisms. This implies that the mechanical processes governing tissue morphogenesis can be delineated into distinct setups that collaboratively control the dynamic behaviors of a single tissue. Yet, the potential interactions or independence among these mechanisms remain unclear.

4. Number videos according to appearance in manuscript (1-2-5-4 is the order used)-

Response: Thank you for the comment. We have renumbered the videos according to their appearance in the manuscript.

5. Add Myo1d, E-cad and Par3 to keywords.

Response: We have included them in the keywords.
