# Supplementary material for: Distinct cellular and junctional dynamics independently regulate the rotation and elongation of the embryonic gut in Drosophila
Source: PLoS Genet. 2024 Oct 7;20(10):e1011422. doi: 10.1371/journal.pgen.1011422 (PMC11486408; doi:10.1371/journal.pgen.1011422)
Supplement: Peer review history

# Peer review: Decision Letter 0

**DOI:** 10.1371/journal.pgen.1011422.r001

**Authors:** Ken M. Cadigan, Gregory P. Copenhaver

**Transfer Alert**

This paper was transferred from another journal. As a result, its full editorial history (including decision letters, peer reviews and author responses) may not be present.

22 Mar 2024

Dear Dr Inaki,

Thank you very much for submitting your Research Article entitled 'Distinct cellular and junctional dynamics independently regulate the rotation and elongation of the embryonic gut in Drosophila' to PLOS Genetics.

The manuscript was fully evaluated at the editorial level and by independent peer reviewers. The reviewers appreciated the attention to an important problem, but raised some substantial concerns about the current manuscript. Based on the reviews, we will not be able to accept this version of the manuscript, but we would be willing to review a much-revised version. We cannot, of course, promise publication at that time.

Should you decide to revise the manuscript for further consideration here, your revisions should address the specific points made by each reviewer. We will also require a detailed list of your responses to the review comments and a description of the changes you have made in the manuscript.  The reviewers all expressed that they require additional explanation of the proposed model and clarification of the experimental definitions of the cell behaviors described in the manuscript.  The authors should consider their concerns carefully and make sure that the revised manuscript addresses these concerns (rather than just clarifying in the point-by-point response).

If you decide to revise the manuscript for further consideration at PLOS Genetics, please aim to resubmit within the next 60 days, unless it will take extra time to address the concerns of the reviewers, in which case we would appreciate an expected resubmission date by email to plosgenetics@plos.org.

If present, accompanying reviewer attachments are included with this email; please notify the journal office if any appear to be missing. They will also be available for download from the link below. You can use this link to log into the system when you are ready to submit a revised version, having first consulted our Submission Checklist.

To enhance the reproducibility of your results, we recommend that you deposit your laboratory protocols in protocols.io, where a protocol can be assigned its own identifier (DOI) such that it can be cited independently in the future. Additionally, PLOS ONE offers an option to publish peer-reviewed clinical study protocols. Read more information on sharing protocols at https://plos.org/protocols?utm_medium=editorial-email&utm_source=authorletters&utm_campaign=protocols

Please be aware that our data availability policy requires that all numerical data underlying graphs or summary statistics are included with the submission, and you will need to provide this upon resubmission if not already present. In addition, we do not permit the inclusion of phrases such as "data not shown" or "unpublished results" in manuscripts. All points should be backed up by data provided with the submission.

While revising your submission, please upload your figure files to the Preflight Analysis and Conversion Engine (PACE) digital diagnostic tool.  PACE helps ensure that figures meet PLOS requirements. To use PACE, you must first register as a user. Then, login and navigate to the UPLOAD tab, where you will find detailed instructions on how to use the tool. If you encounter any issues or have any questions when using PACE, please email us at figures@plos.org.

PLOS has incorporated Similarity Check, powered by iThenticate, into its journal-wide submission system in order to screen submitted content for originality before publication. Each PLOS journal undertakes screening on a proportion of submitted articles. You will be contacted if needed following the screening process.

To resubmit, use the link below and 'Revise Submission' in the 'Submissions Needing Revision' folder.

We are sorry that we cannot be more positive about your manuscript at this stage. Please do not hesitate to contact us if you have any concerns or questions.

Yours sincerely,

Ken M. Cadigan, PhD

Academic Editor

PLOS Genetics

Gregory P. Copenhaver

Editor-in-Chief

PLOS Genetics

Reviewer's Responses to Questions

**Comments to the Authors:**

**Please note here if the review is uploaded as an attachment.**

Reviewer #1: Major Comment

This manuscript follows up on the authors' previous works on the chiral rotation of the epithelial tube, the Drosophila hindgut. They previously showed that the rotation involves two steps. First, the epithelial cells in the gut tube, which itself is straight, take a chiral cell shape. In the second elongation step, the tube rotates while the cell shape asymmetry is resolved. In this work, the authors focused on the cell behavior during the tube elongation step. They attempted to dissect elements of cell shape change and their contribution to the tube rotation. Using genetic and pharmacological perturbation experiments, quantitative measurement, and mathematical modeling, the authors suggested two cell behavior elements, “cell sliding” and “convergent extension,” contribute differently to the tube rotation. I should raise three issues and ask the authors to resolve them.

1. To argue the independence of the two elements, “cell sliding” and “convergent extension,” each value should represent distinct, independent cell properties. Cell sliding was defined as shifting the left-right position of a pair of adjacent cell nuclei. Formally, the shift can result from a simple cell position change with a conserved cell interface. Alternatively, the shift may involve the sliding of cell boundaries with changes in cell interface relationship (intercalation). As the authors demonstrated, cell intercalation is the major driver of convergent extension. Since cell intercalation could also be involved in cell sliding, the two values cannot be considered independent. For the argument to stand, the authors should provide evidence that the two values are separate measures of cell behavior and present them before they start arguing about possible relationships of the values.

2. The authors measured the tube length from 3D stacks of live imaging data. If I understand correctly, the length value was taken from the straight line between the anterior-most edge of the tube and the author-defined posterior starting point (Fig. S1). The ideal measure for the tube length should be the length of the tracked tube lumen, which is curved in 3D space. I need clarification on the rationale of using straight lines to represent the length of curved and rotated tubes.

3. The authors compared the tube elongation behavior in control, E-cad mutant and MyoID overexpression. Those genetic perturbations can also influence the first step of cell chirality before cell elongation. If this is the case, the elongation movement started from different starting conditions. How can the authors argue that E-cad and MyoID overexpression differently affect cell behavior at the time of elongation, not before?

Other Comments

Abstract. “simultaneous” was used 6 times.

Line 24. “we demonstrated that independently regulated cellular dynamics can simultaneously cause shape changes in a mechanical system.”　The sentence does not seem to provide meaningful information.

Line 37 “Organ shape is closely related to its functions; therefore, it needs to be precisely controlled during development1.” Difficult sentence. Maybe “Organ shapes are optimized to execute their function.”

Line 38 What is indicated by “Epithelial morphology” here?

Abstract and Introduction. The words “simultaneous” and “coordinate” are used to describe the relationship of more than two events (or parameters). What was compared was inconsistent. In the introduction, cellular vs molecular mechanisms were compared. In the abstract, anteroposterior elongation and left-right shifting are compared.

Line 202 “This transition encourages these epithelial cells to intercalate with each other,“ encourage may be replaced by drive, promote, etc.

Reviewer #2: The authors presented an intriguing study on the simultaneous shape changes in Drosophila embryonic hindgut morphogenesis, focusing on cell sliding and convergent extension. The integration of genetic analysis, live imaging, and mathematical modeling offers valuable insights into complex organ formation. However, there are several areas where clarification and additional information are required to support the findings and conclusions more robustly.

1. Clarification on Contractions and Equations (P17 L275):

Please clarify the relationship between contractions with chirality and anterior-posterior/distal-proximal anisotropy and Equations 4 and 5. How are these phenomena represented within the mathematical model?

2. Reference to Equations for Axial and Radial Expansion (P17 L282):

Is the consideration 'axial and radial expansion and contraction of the hindgut' referring to Equation 2?

3. Question on Two Oriented Angles (P17 L287):

In the section discussing 'edge tension biased at two oriented angles' (Fig4E), could you specify what is meant by 'two oriented angles'? The figure appears to depict only one angle. Additionally, could you explain the concept of 'left-right-biased contraction' mentioned in the caption, and how it relates to the contractions with chirality and anterior-posterior/distal-proximal anisotropy introduced earlier?

4. Clarification on Cell Chirality Introduction (P18 L288):

The text states 'Cell chirality was introduced as cell shape strain under the initial condition.' Is this referring to the cell shapes shown in Figure 4G?

5. Chirality in E-cad-mutant Cells and Simulations (P18):

The authors mentioned that cell chirality was found to be abolished in E-cad-mutant cells (p8 L120). In the simulations under E-cad-null conditions (Fig4I), is chirality assumed initially similar to other simulations? If so, why doesn't Fig4I seem to show the expected disappearance of chirality followed by cell sliding?

6. Frequency of Cell Intercalation in Simulations vs. Experimental Observations:

It's noted that cell intercalation occurs at approximately a 10% frequency in experiments. Do the simulations replicate this frequency, particularly in explaining the elongation rate? It would be important to verify whether the model's assumptions about cell intercalation frequency align with experimental observations to support the model's validity.

7. Model's Representation of Cell Boundary Contractions (P18 L292):

It is unclear which parts of the equations correspond to 'cell boundary contractions with chirality or anterior–posterior/distal–proximal anisotropy' as they are introduced separately in the model. Could you provide further explanation in Materials and Methods?

8. Directionality and Figure References (P18 L295, L297, L299):

Regarding 'in the counterclockwise direction' (Figure 4G), should this refer to Fig4H? Given the right-hand coordinate system, would the direction be clockwise if the z-axis orientation is considered?

It seems there might be confusion in figure references: Fig4H seems to be referred to as Fig4I, and Fig4I as Fig4J. Could you clarify these references?

9. Request for Detailed Model Explanation and Equation Clarification:

The explanation of the model and its underlying equations appears overly simplified, making it difficult to grasp the essential details. A more detailed explanation is requested.

Specifically, Equation 2 seems to lack boundary condition terms that would ensure equilibrium states conform to physical laws, such as Laplace's law.

For the circumferential direction, the equilibrium state should balance the external pressure difference P across the tube's wall and the circumferential tension, determining the radius R, (represented here as the circumferential length Lx). The equation might be represented as:

xi dLx/dt = -Lx sigma_xx + Lx^2 P/h where h is the cell height (along apicobasal axis).

Similarly, for the axial direction, considering the tube is connected to surrounding tissues, the boundary stress could be represented as sigma_n, leading to:

xi dLy/dt = -Ly(sigma_yy – sigma_n)

These considerations raise questions about the stability of cylindrical shapes under the authors' original equations, particularly if they do not reconcile with Laplace's law at equilibrium, suggesting that the cylindrical shape visualized in Fig4 might not be stable under the author’s proposed condition (P=0).

The authors' original formulation for the circumferential direction seems to lack an explicit representation of these dynamics, potentially overlooking how the equilibrium tube radius R (Lx) is determined by the balance of forces.

It is believed that this could have a significant impact on convergent extension.

Therefore, additional discussion on this aspect would be highly beneficial.

Moreover, the application of isotropic linear elasticity, as possibly assumed in deriving Equation 3, to conditions leading to plastic deformations like convergent extension warrants further discussion.

Also, the role and significance of zeta in Equation 4 are not described. Clarification on its meaning and the rationale for not assuming linear elasticity (zeta = 0) would provide valuable insights for readers.

Reviewer #3: Inaki, Matsuno and colleagues study the cell biology of the development of the gut in the Drosophila embryo, using wt and mutant strains. This model system is particularly interesting and relevant as it combines asymmetric rotation by cell sliding and AP elongation by convergent extension (CE). The authors impressively demonstrate that both processes are independently regulated by the actin-based molecular motor MyoID and the adhesion protein E-cad on the one hand (cell sliding) and the cell polarity regulator Par3.

The results are well presented, tightly controlled and of high quality. This is particularly

true for the movies. Although I tried hard I did not find absolutely necessary additional experiments to be performed.

Due to lack of competency, I could not judge the model.

What I, however, truly missed is a proper discussion. What is presented is in large parts a repetition of the results. How do these results relate to the published literature? What do they mean in an evolutionary context?

-minor language issues (line 16 add ‚responsible’ before epithelium; add Myo1D to Keywords; non-drosophilists may have problems to follow the name of mutants (i.e. shg for E-cad); line 152: occasionaly = almost 50%

First paragraph of Intro: which ‘hypothesis’ is eluded to (line 46)?

Number videos according to appearance in manuscript (1-2-5-4 is the order used)-

Add Myo1d, E-cad and Par3 to keywords.

**********

**Have all data underlying the figures and results presented in the manuscript been provided?**

Large-scale datasets should be made available via a public repository as described in the *PLOS Genetics* data availability policy, and numerical data that underlies graphs or summary statistics should be provided in spreadsheet form as supporting information.

Reviewer #1: Yes

Reviewer #2: None

Reviewer #3: Yes

**********

PLOS authors have the option to publish the peer review history of their article (what does this mean?). If published, this will include your full peer review and any attached files.

If you choose “no”, your identity will remain anonymous but your review may still be made public.

**Do you want your identity to be public for this peer review?** For information about this choice, including consent withdrawal, please see our Privacy Policy.

Reviewer #1: No

Reviewer #2: No

Reviewer #3: **Yes: **Martin Blum

# Author comment: Author response to Decision Letter 0

**DOI:** 10.1371/journal.pgen.1011422.r002

23 Jul 2024

Attachment Submitted filename: 240718_response to revierwes.docx

# Peer review: Decision Letter 1

**DOI:** 10.1371/journal.pgen.1011422.r003

**Authors:** Ken M. Cadigan, Gregory P. Copenhaver

6 Sep 2024

Dear Dr Inaki,

We are pleased to inform you that your manuscript entitled "Distinct cellular and junctional dynamics independently regulate the rotation and elongation of the embryonic gut in Drosophila" has been editorially accepted for publication in PLOS Genetics. Congratulations!

Before your submission can be formally accepted and sent to production you will need to complete our formatting changes, which you will receive in a follow up email. Please be aware that it may take several days for you to receive this email; during this time no action is required by you. Please note: the accept date on your published article will reflect the date of this provisional acceptance, but your manuscript will not be scheduled for publication until the required changes have been made.

Once your paper is formally accepted, an uncorrected proof of your manuscript will be published online ahead of the final version, unless you’ve already opted out via the online submission form. If, for any reason, you do not want an earlier version of your manuscript published online or are unsure if you have already indicated as such, please let the journal staff know immediately at plosgenetics@plos.org.

In the meantime, please log into Editorial Manager at https://www.editorialmanager.com/pgenetics/, click the "Update My Information" link at the top of the page, and update your user information to ensure an efficient production and billing process. Note that PLOS requires an ORCID iD for all corresponding authors. Therefore, please ensure that you have an ORCID iD and that it is validated in Editorial Manager. To do this, go to ‘Update my Information’ (in the upper left-hand corner of the main menu), and click on the Fetch/Validate link next to the ORCID field.  This will take you to the ORCID site and allow you to create a new iD or authenticate a pre-existing iD in Editorial Manager.

If you have a press-related query, or would like to know about making your underlying data available (as you will be aware, this is required for publication), please see the end of this email. If your institution or institutions have a press office, please notify them about your upcoming article at this point, to enable them to help maximise its impact. Inform journal staff as soon as possible if you are preparing a press release for your article and need a publication date.

Thank you again for supporting open-access publishing; we are looking forward to publishing your work in PLOS Genetics!

Yours sincerely,

Ken M. Cadigan, PhD

Academic Editor

PLOS Genetics

Gregory P. Copenhaver

Section Editor

PLOS Genetics

www.plosgenetics.org

Twitter: @PLOSGenetics

----------------------------------------------------

Comments from the reviewers (if applicable):

Reviewer's Responses to Questions

**Comments to the Authors:**

**Please note here if the review is uploaded as an attachment.**

Reviewer #1: The author responded to my comments in a reasonable manner. Now that I feel that the manuscript is appropriate for PLOS Genetics.

Reviewer #2: The authors have responded adequately to my comments.

Reviewer #3: Inaki, Matsuno and colleagues have addressed my critics and comments in a truly satifactory manner. I therefore recommend now publication as is.

**********

**Have all data underlying the figures and results presented in the manuscript been provided?**

Large-scale datasets should be made available via a public repository as described in the *PLOS Genetics* data availability policy, and numerical data that underlies graphs or summary statistics should be provided in spreadsheet form as supporting information.

Reviewer #1: Yes

Reviewer #2: None

Reviewer #3: Yes

**********

PLOS authors have the option to publish the peer review history of their article (what does this mean?). If published, this will include your full peer review and any attached files.

If you choose “no”, your identity will remain anonymous but your review may still be made public.

**Do you want your identity to be public for this peer review?** For information about this choice, including consent withdrawal, please see our Privacy Policy.

Reviewer #1: No

Reviewer #2: No

Reviewer #3: **Yes: **Martin Blum

----------------------------------------------------

**Data Deposition**

If you have submitted a Research Article or Front Matter that has associated data that are not suitable for deposition in a subject-specific public repository (such as GenBank or ArrayExpress), one way to make that data available is to deposit it in the Dryad Digital Repository. As you may recall, we ask all authors to agree to make data available; this is one way to achieve that. A full list of recommended repositories can be found on our website.

The following link will take you to the Dryad record for your article, so you won't have to re‐enter its bibliographic information, and can upload your files directly: 

http://datadryad.org/submit?journalID=pgenetics&manu=PGENETICS-D-24-00075R1

More information about depositing data in Dryad is available at http://www.datadryad.org/depositing. If you experience any difficulties in submitting your data, please contact help@datadryad.org for support.

Additionally, please be aware that our data availability policy requires that all numerical data underlying display items are included with the submission, and you will need to provide this before we can formally accept your manuscript, if not already present.

----------------------------------------------------

**Press Queries**

If you or your institution will be preparing press materials for this manuscript, or if you need to know your paper's publication date for media purposes, please inform the journal staff as soon as possible so that your submission can be scheduled accordingly. Your manuscript will remain under a strict press embargo until the publication date and time. This means an early version of your manuscript will not be published ahead of your final version. PLOS Genetics may also choose to issue a press release for your article. If there's anything the journal should know or you'd like more information, please get in touch via plosgenetics@plos.org.

# Editor report: Acceptance letter

**DOI:** 10.1371/journal.pgen.1011422.r004

**Authors:** Ken M. Cadigan, Gregory P. Copenhaver

1 Oct 2024

PGENETICS-D-24-00075R1 

Distinct cellular and junctional dynamics independently regulate the rotation and elongation of the embryonic gut in Drosophila 

Dear Dr Inaki, 

We are pleased to inform you that your manuscript entitled "Distinct cellular and junctional dynamics independently regulate the rotation and elongation of the embryonic gut in Drosophila" has been formally accepted for publication in PLOS Genetics! Your manuscript is now with our production department and you will be notified of the publication date in due course.

The corresponding author will soon be receiving a typeset proof for review, to ensure errors have not been introduced during production. Please review the PDF proof of your manuscript carefully, as this is the last chance to correct any errors. Please note that major changes, or those which affect the scientific understanding of the work, will likely cause delays to the publication date of your manuscript. 

Soon after your final files are uploaded, unless you have opted out or your manuscript is a front-matter piece, the early version of your manuscript will be published online. The date of the early version will be your article's publication date. The final article will be published to the same URL, and all versions of the paper will be accessible to readers.

Thank you again for supporting PLOS Genetics and open-access publishing. We are looking forward to publishing your work! 

With kind regards,

Jazmin Toth

PLOS Genetics

On behalf of:

The PLOS Genetics Team

Carlyle House, Carlyle Road, Cambridge CB4 3DN | United Kingdom

plosgenetics@plos.org | +44 (0) 1223-442823

plosgenetics.org | Twitter: @PLOSGenetics
